# Fabrication, Characterization, and Sensor Applications of Polymer-Based Whispering Gallery Mode Microresonators

**DOI:** 10.1021/acssensors.5c00057

**Published:** 2025-07-31

**Authors:** Jarosław Mazuryk, Piotr Paszke, Dorota A. Pawlak, Włodzimierz Kutner, Piyush Sindhu Sharma

**Affiliations:** † Institute of Physical Chemistry, 119463 Polish Academy of Sciences, Kasprzaka 44/52, 01-224 Warsaw, Poland; ‡ Faculty of Chemistry, 49605 University of Warsaw, Pasteura 1, 02-093 Warsaw, Poland; § ENSEMBLE^3^ sp. z o. o., Wolczynska 133, 01-919 Warsaw, Poland; ∥ Modified Electrodes for Potential Application in Sensors and Cells Research Team, Institute of Physical Chemistry, Polish Academy of Sciences, 01-224 Warsaw, Poland; ⊥ The Faculty of Mathematics and Natural Sciences, School of Sciences, Cardinal Stefan Wyszynski University in Warsaw, Wóycickiego 1/3, 01-938 Warsaw, Poland; # Functional Polymers Research Team, Institute of Physical Chemistry, Polish Academy of Sciences, Kasprzaka 44/52, 01-224 Warsaw, Poland; ▲ Bio & Soft Matter, Institute of Condensed Matter and Nanosciences, Université Catholique de Louvain, 1 Place Louis Pasteur, 1348 Louvain-la-Neuve, Belgium

**Keywords:** bio- and chemosensing, whispering gallery mode resonator (WGMR), microfabricated optical microbubble and optofluidic ring resonator, polymer optical fiber (POF) and photonics, polymer resonator, molecularly imprinted polymer (MIP), all-polymer WGMR, (polymer shell)-(inorganic core) composite WGMR, microresonator

## Abstract

The present article critically reviews the fabrication, characterization, and sensor applications of polymer-based whispering gallery mode resonators (WGMRs). Those resonators utilize continuous internal light reflections along curved surfaces to produce sharp resonance peaks influenced by the resonator’s geometry, which appeared effective for high-sensitivity optical sensing. Polymer-based WGMRs leverage unique polymer characteristics to enhance sensor performance through parameters like quality factor (QF), free spectral range (FSR), resonance mode shifts, polarization modes, bulk refractive index (RI), sensitivity per refractive index unit (RIU), and thermo-optic effects. All-polymer WGMRs, i.e., resonators entirely made from polymers, offer design flexibility, biocompatibility, low thermal conductivity, and integration capabilities for high sensitivity, detectability, and selectivity. Polymer-coated optical fiber WGMRs improve light–material interaction, support advanced composites, integrate with microfluidics for on-chip diagnostics, and enable remote, multiplexed sensing. (Polymer shell)-(inorganic core) composite-functionalized WGMRs combine the high QFs of inorganic materials with polymers’ flexibility and functionalization, providing synergistic optical properties, enhanced sensitivity, detectability, and stability. These advancements make polymer-based WGMR sensors promising for biomedical diagnostics, environmental pollution monitoring, and industrial process control. Future research will presumably optimize fabrication techniques, explore novel polymers, and integrate advanced signal processing for real-time analysis, connected with the Internet-of-Things (IoT) and cloud databases to revolutionize optical and photonic sensing platforms.

## Graphical abstract

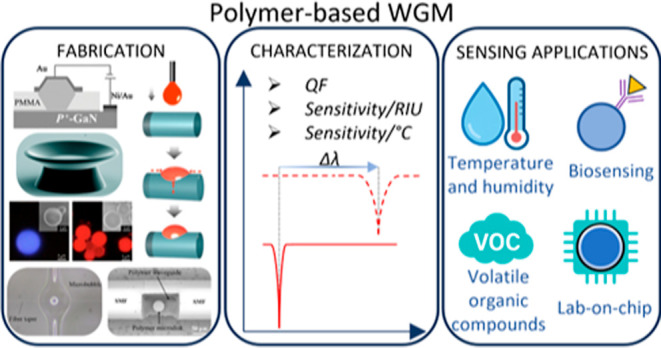

Whispering gallery mode resonators (WGMRs) have emerged as powerful tools for high-sensitivity optical sensing, leveraging the unique optical phenomenon where light circulates along the periphery of a curved surface via total internal reflection. Whispering gallery modes (WGMs) refer to the optical modes that circulate within a resonator, typically of spherical or cylindrical geometry, due to continuous total internal reflections at the boundary. This phenomenon leads to the formation of resonances characterized by sharp peaks in the transmission or reflection spectrum. WGMRs resonate when light, after completing a trip around the resonator cavity, constructively interferes with the pumping wave.^1–4^ WGMRs can be categorized based on their geometries: Spherical WGMRs are among the most widely studied configurations because of their high symmetry and simple fabrication.^5–10^ Their geometry naturally supports the formation of high-quality WGMs with minimal scattering losses, resulting in exceptionally high values of quality factors (QFs). The optical confinement in spherical structures is predominantly determined by total internal reflection at the surface, making their performance highly sensitive to material purity and surface smoothness. WGMRs serve as a fundamental platform for understanding light–matter interactions in curved dielectric structures. Although the spherical geometry is most commonly employed due to its simplicity and high symmetry, alternative shapes, including disks^11–13^ and toroids,^14,15^ are also frequently utilized, offering additional degrees of freedom for tailoring modal properties, enhancing mechanical stability, and facilitating integration into photonic systems.^11^ Moreover, capillary-based^16,17^ and bubble-shaped^18,19^ resonators have been devised. These resonators, due to their hollow cylindrical geometries and thin-walled hollow spheres fabricated from capillaries, enable efficient coupling with fluids,^20^ making them highly suitable for optofluidic applications and refractive index (RI) sensing.^17^ Both geometries facilitate easy analyte delivery, expanding the scope of sensing strategies utilizing WGMRs.

In general, WGMRs can be characterized by several key parameters that apply to all types of resonators.^2,21^ (i) The QF is a key parameter that describes the efficiency of light confinement within the resonator. It is defined as the ratio of the energy stored to the energy lost per cycle. A high QF indicates that the resonator can trap light for longer durations, allowing for its multiple circulations around the cavity with minimal energy loss. This trapping results in sharp resonance peaks and enhances the interaction between the light and the material within the resonator, making high-quality WGMR ideal for applications in lasers and sensing.^21^ (ii) Free spectral range (FSR) determines the separation between adjacent resonant modes in the frequency domain. FSR is inversely proportional to the resonator’s circumference or diameter, influencing the spectral resolution and multiplexing capabilities.^21^ (iii) Mode volume is a parameter that describes the spatial extent over which the optical field of a given mode is distributed. Smaller values of mode volume are desirable because they enhance light–matter interactions. In the context of WGMRs, achieving a small mode volume while maintaining a high QF is crucial for sensing and lasing applications. A reduced mode volume results in a stronger confinement of the optical field, increasing its intensity near the resonator surface.^21^ (iv) Sensitivity per refractive index unit (RIU) quantifies the shift of the resonance wavelength of a WGMR in response to a unit change in the RI of the surrounding medium. It is typically expressed in nm/RIU and is a critical parameter for evaluating the performance of optical sensors based on WGMR structures. It is crucial to determine the sensor’s limit of detection (LOD) and linear dynamic concentration range.^2^ (v) The thermo-optic effect results from temperature-induced changes in the RI, thus providing a quantitative measure of environmental parameters.^2^

Polymer-based WGMRs, whose sensing properties are summarized in Table 1, represent a burgeoning field that integrates polymers with resonators to exploit their unique optical, mechanical, and chemical features. These resonators can broadly be categorized into several types based on their construction and the material used. Principal types include all-polymer WGMRs, polymer film-coated optical fiber (OF) WGMRs, and (polymer shell)-(inorganic core) composite-functionalized WGMRs.

**Table 1:** Sensing Properties of Whispering Gallery Mode Resonators (WGMRs)

polymer-based WGMRs	quality factor (QF)	sensing properties	ref
all-polymer WGMRs			
poly(methyl methacrylate) (PMMA) WGMRs prepared by mechanical machining and equatorial ring-polishing	3 × 10^5^, 4 × 10^7^	n/a	^30^
a dyed-doped SU-8 microbottle WGMR laser devised by droplet self-assembly	320, 1460, 2200	n/a	^31^
SU-8 WGMRs doped with LDS698, rhodamine B, rhodamine-6G (R6G), and rhodamine 123	10^5^ to 10^6^	n/a	^32^
(2–10) μm in diameter microspheres (MSs) formed from self-assembled π-conjugated alternating copolymers	100–600	n/a	^10^
Self-assembled π-conjugated alternating poly[(9,9-dioctylfluorene-2,7-diyl)-(5-octylthieno[3,4-*c*]pyrrole-4,6-dione-1,3-diyl)-based MSs	∼10^4^	n/a	^33^
Arrays of SU-8 suspended-disk WGMRs coupled to tapered OFs	6.4 × 10^3^, 4.9 × 10^3^	n/a	^34^
rod-shape WGMR composite of π-conjugated poly[2,5-bis(20,50-bis(200-ethylhexyloxy)phenyl)-*p*-phenylenevinylene] and a UV transparent 1,4-cyclohexanedimethanol divinyl ether matrix	n/m	n/a	^35^
R6G-doped PDMS-based in-elastomer droplet WGMRs fabricated by a needle-dipping method	∼10^3^	n/a	^36^
Inkjet printing-based layer stacking-fabricated in-spot hyperbranched TZ-001/TZ002 LDS798 dye-doped droplet microdisks	∼10^7^	n/a	^37^
all-optical, microfluidic poly[(9,9-dioctylfluorenyl-2,7-diyl)-*alt*-*co*-(1,4-benzo-(2,10,3)thiadiazole)]-based laser diode-pumped polymer WGMR laser	5.17 × 10^3^	n/a	^17^
droplet-inspired pressure-modeled and temperature-insensitive NOA65-based MS WGMR	10^5^	*p* (0–27 N)-*T* (26–32 °C)-wavelength red-shift and RIs changes (10^–4^ to ∼10^–5^), sensitivity: 0.006 nm/°C	^38^
a self-assembly SU-8 WGMR packaged in a cured PDMS support	∼10^4^	sensitivity: 120 pm/°C	^39^
microfluidic PMMA-based pyrromethene 597-doped microgoblet laser for refractometric sensing of glycerol–water solutions	∼10^5^	sensitivity: 10.56 nm/RIU	^15^
arrayed PMMA MSs for temperature sensing in the range of 25–35.8 °C	10^7^–10^9^	sensitivity: 0.001 nm/K	^22^
pentanoic acid binding-induced swelling of pillared SU-8 microdisk WGMR	5 × 10^4^	sensitivity: 23 pm/ppm, LOD: 0.6 ppm	^26^
acetone-sensitive π-conjugated poly(9,9-dioctylfluorene-*alt*-benzothiadiazole)-based MS WGM microlaser fabricated by emulsion-solvent	0.5–1.5 × 10^3^	Sensitivity: 0.21 nm/ppm, LOD: 90 ppb	^40^
a trichromatic single-mode laser MR based on R6G-doped polymer self-assembled in 3D-curved microcavities for the detection of acetic acid	∼10^5^	sensitivity: 186.5 pm/min, LOD: 5 min	^27^
PS MS WGMRs doped with β-cyano-appended oligo(*p*-phenylenevinylene) prepared by miniemulsion for detecting vapors	1080	sensitivity: 1.49 nm/μL	^41^
SU-8-based microfluidic label-free WGMR sensor of glucose fabricated by a hybrid femtosecond laser micromachining	5 × 10^3^	sensitivity: 61 nm/RIU, LOD: 0.0048 RIU	^42^
liposome-incorporating layer-by-layer (LbL) assemblies of cationic (branched polyethylenimine-based) glycopolymers and anionic (sulfuric and sialic acid–based) glycopolymers containing *N*-acetylgalactosamine, lactose, and maltose deposited on PS sulfonate and poly(allylamine hydrochloride) precoated WGMR sensor particles	n/m	n/m	^14^
polymer microcavities written by two-photon polymerization (hybrid Zr/Si sol–gel)	1.48 × 10^5^	n/a	^43^
femtosecond-laser direct writing of polymer WGMRs via two-photon polymerization	1 × 10^5^	n/a	^44^
asymmetric polymer microdisk resonators fabricated via two-photon polymerization	1.19 × 10^5^	n/a	^45^
a laser-written 4D optical microcavity for advanced biochemical sensing in an aqueous environment	4 × 10^4^	sensitivity: 457 nm/RIU	^46^
polymer optical fibers (POFs)			
Ag nanowires incorporating R6G-doped PMMA optical fiber (POF)	>10^4^	n/m	^47^
PS MS-encapsulating Ge-doped silica core single-mode microstructured OFs suspended within three hollow channels	∼2.2 × 10^3^	n/m	^48^
Au nanorod-coupled Rhodamine 101-doped PMMA WGMRs	n/m	n/m	^49^
R6G-doped PMMA microfiber POF WGMRs	∼6 × 10^3^	n/m	^42^
quasi-3D coupled WGM microcavities consisting of intersected self-assembly POFs based on disodium 4,4′-bis(2-sulfonatostyryl)biphenyl, polyvinyl alcohol, and cetylmethylammonium bromide	5.5 × 10^3^	n/m	^50^
a hollow-core all-in-silica fiber internally integrated polymer microdisk WGMR, printed by femtosecond laser-induced two-photon polymerization	2.3 × 10^3^	temperature range: 26–60 °C, Sensitivity: –95 pm/°C, RH range: 30–90% sensitivity: 54 pm/% RH	^11^
a B-type starch-based 4-[*p*-(dimethylamino)styryl]-1-methylpyridinium POF microlaser	∼10^3^	n/m	^24^
a microstructured WGM PS MS-attached POF resonator was devised for self-referenced sensing of neutravidin	n/m	detecting 25, 50, 100, 400 nM neutravidin	^51^
A 3D prefabricated on-chip goblet-shaped passive PMMA WGMRs fabricated by dip-pen nanolithography for optofluidic sensing of streptavidin	n/m	50 nanomoles of streptavidin	^52^
thin-walled microfabricated optofluidic ring resonators (μOFRRs) and optical microbubble resonators (OMBRs) for enabling the sensitive detection of fluids	355	sensitivity: 2510 nm/RIU, LOD: 1.6 × 10^–5^ RIU	^53^
a monolithic on-chip PDMS film-coated μOFRR sensor for volatile organic compounds	1.15 × 10^4^	sensitivity: 1 pm/(mg/m^3^)	^54^
a PDMS film-lined μOFRR combined with an Si-microfabricated 2D gas chromatographic microsystem for sensing organic vapors	n/m	LOD: 8–19 ng	^55^
PDMS/SU-8 WGM μOFRR fabricated by UV lithography-based 3D printing for (horseradish peroxidase-streptavidin)-based enzyme-linked immunosorbent assay-sensing of the vascular endothelial growth factor	9.8 × 10^3^, 7 × 10^3^, 5.8 × 10^3^	LOD: 17.8 fg/mL	^56^
polydopamine-functionalized liquid crystal (LC) molecules-modified HGMS-embedded capillary-fiber probe containing immobilized (fluorescein isothiocyanate)-labeled cardiac troponin (FITC-cTnI-C) antibody for sensing cTnI-C	n/m	LOD: 0.59 ng/mL	^17^
LC (dimethyloctadecyl[3-(trimethoxysilyl)propyl]ammonium chloride)-containing HGMS for sensing cTnI-C	2.63 × 10^3^	LOD: 1.103 ng/mL	^57^
Waveguide-coupled polymer microring resonator in a D-shaped fiber	4.86 × 10^3^	sensitivity: –193 pm/°C	^58^
(polymer shell)-(inorganic core) composite conjugated WGMRs			
replica-modeling of PDMS- and Vicast-coated ultrahigh-quality silica microtoroid arrays	PDMS: 2 × 10^6^ Vicast: 5 × 10^6^	n/m	^59^
PMMA- and PS-coated ultrahigh-quality silica microtoroids	>10^7^	n/m	^60^
multilayer dielectric Si-core MS WGMRs, fabricated based on (i) single-PDMS-layer spheres, (ii) multilayered PDMS cores coated with BaTiO_3_ and PDMS films, and (iii) Si cores coated with a thin layer of uncured PDMS base coatings	10^6^	1.7 pm (kV/m), 2.5 pm (kV/m), and 0.2 pm (V/m)	^61^
a micrometer composite of SiO_2_ microparticle cores coated with a conjugated poly(1-vinylpyrrolidone-*co*-vinyl shell, prepared via seeded Knoevenagel dispersion polymerization	n/m	n/m	^62^
silica WGMR surfaces with covalently bound fluorescein isothiocyanate-labeled poly(ethylene glycol) (PEG) spacers	>10^6^	n/m	^63^
PEG film-coated SiO_2_ WGMRs conjugated with the avidin–biotin analyte-recognition element system	>10^6^	(20–30)-pm shift in the 100–1000 μg/mL avidin	^64^
Ellipsoidal silica OMBRs, coated with polyhexamethylene biguanide films via filling and sintering, for sensing carbon dioxide	7.33 × 10^4^	sensitivity: 0.46 pm/ppm, LOD: 50 ppm	^65^
swellable pH-sensitive hydrogel-embedded *N*-isopropylacrylamide particles layered on silica hollow bottle WGMRs for optical frequency-shift refractometric pH sensing	n/m	sensitivity: 33 nm/RIU 0.06 pH unit	^66^
optical microbubble resonator (OMBR) of diameter and wall thickness of ∼256 and ∼1.7 μm, respectively, was silanized using 3-glycidoxypropyltrimethoxysilan-functionalized OMBR internally coated with cTnI-C antibody for sensing cTnI-C	∼1.5 × 10^5^	sensitivity: 6.3 nm/RIU, LOD: 0.4 ag/mL	^49^
a heterostructured microlaser diode devised from a hexagonal ZnO microrod incorporated in an interface PMMA matrix and integrated with a p(+)-GaN semiconducting substrate	550	n/m	^67^
a self-rolled-up oxide tubular silica-supported nanocomposite consisted of Al_2_O_3_/Y_2_O_3_/ZrO_2_/Al_2_O_3_ WGM microcavity coated with poly(acrylic acid)/poly(ethylenimine) polymers designed in a polymer/oxide/oxide architecture for sensing environmental RH	n/m	sensitivity: 130 pm/RH unit	^68^
electrotunable WGM microlaser based on a 0.7Pb(Mg_1/3_Nb_2/3_)O_3_-0.3PbTiO_3_ piezoelectric crystal combined with a poly[9,9-dioctylfluorenyl-2,7-diyl] microring cavity devised by employing inkjet printing	3.28 × 10^3^, 3.53 × 10^3^, 4.62 × 10^3^	n/m	^69^
self-assembled π-conjugated poly[(9,9′-dioctyl-9*H*-fluorene-2,7-yl)-5,5′-(2,2’:6′,2“-terpyridine) alternating Eu^3+^-copolymer MSs	n/m	n/m	^70^
a microtoroid WGMR with an Nd micromagnet glued to the film of the supporting UV curable polymer for pressure-induced deformation of the encapsulating polymer, causing changes in polymer RI	∼10^6^	sensitivity: 880 pT/Hz^1/2^	^18^
single-crystalline 1D microwire and 2D microplate Cd^2+^-noded MOFs containing 1,1,2,2-tetrakis(4′-(pyridine-4-yl)-[1,1′-biphenyl]-4-yl)-ethene ligand-based microlasers	∼10^3^	n/m	^71^
self-assembly dye-doped SWCNTs onto PS MS WGMRs employed to fabricate near-infrared semiconducting nanolasers	(3.5–4) × 10^3^	n/m	^72^
a tapered single-mode OF-derived MSs coated with a film of inorganic silane-based FITC-selective molecularly imprinted polymers (MIPs), prepared by sol–gel transition onto either silica-on-silicon wafers or onto silica MSs via manual or automated dip coating	1.243 × 10^6^	3.62–4.97 × 10^19^ FITC molecules	^73^
6-Carboxyfluorescein-labeled 20-mer ssDNA-coated silica microtoroids designed for detecting Cy5-labeled DNA complement hybridization	2.2 × 10^7^	sensitivity: 1 nM - 2 μM	^74^
Zr-doped silica sol–gel layers-functionalized silica microtoroids designed for the optical enhancement of the Raman–Kerr effect	Zr-free: 1.34 × 10^8^ Zr-doped: 1.52 × 10^7^	a Raman efficiency increase from 0.027 to 0.414%, a Raman threshold decrease from 4.19 to 0.82 mW	^75^

All-polymer WGMRs are resonators fabricated entirely from polymeric materials. Typically, they are manufactured by spin-coating, soft lithography, or direct laser writing. All-polymer WGMRs feature the following properties. (i) Polymers offer flexibility in the design of WGMRs, allowing shaping into various geometries and sizes, facilitating customized resonator designs for dedicated applications.^22^ (ii) The RI and optical properties of polymers can suitably be adjusted by modifying their composition or doping with additives, thus enabling precise control over the WGMR’s spectral characteristics.^23^ (iii) Several polymers are biocompatible, making all-polymer WGMRs appropriate for biosensing applications without adverse reactions in biological environments.^24,25^ (iv) Compared to inorganic materials like silica, the thermal conductivity of polymers is generally lower, which can be beneficial in applications where thermal isolation is important.^26^ (v) Polymers can be integrated with other materials to combine their advantages. For instance, (polymer shell)-(metal core) composites can enhance the sensitivity of WGMRs by harnessing plasmonic effects.^6^ (vi) Miniaturization and integration into microfluidic chips with polymer film-coated OFs enable on-chip sample treatment and preprocessing. The fabrication of multiple-polymer film-coated OFs in a networked configuration allows for the construction of multiplexed sensing arrays for simultaneous sensing of many analytes.^11,27^ This integration enhances the functionality of WGMRs for point-of-care (POC) diagnostics and environmental monitoring. (vii) The properties of polymer coatings can dynamically be altered in response to external stimuli (e.g., changes in temperature, pressure, or chemical environment), enabling real-time monitoring and adaptive sensing capabilities.^28^ (viii) Finally, advances in fabrication techniques allow for the seamless integration of (polymer shell)-(inorganic core) structures, enabling the fabrication of advanced sensing systems that result in improved sensitivity, detectability, and functionality.^29^

## Polymer Whispering Gallery Mode Resonators

### All-Polymer Whispering Gallery Mode Resonators

All-polymer WGMRs are structures in which all components of the resonator are made from polymer materials. Unlike polymer-conjugated resonators (e.g., hybrid silica–polymer resonators), in all-polymer WGMRs, traditional resonator materials, including glass and quartz, are completely replaced by polymers. Several methods have been developed to produce high-performance all-polymer WGMRs, each contributing specific advantages in terms of optical quality and structural precision.

#### Fabrication Methods of All-Polymer Whispering Gallery Mode Resonators

##### Mechanical Machining

Mechanical machining and equatorial ring-polishing methods were employed to fabricate transparent, crystalline, low-loss poly­(methyl methacrylate) (PMMA) WGMRs. The QFs of these PMMA resonators were investigated in two spectral ranges. Under excitation in the near-infrared range (1470–1580 nm), the QF was limited by material absorption and reached 3 × 10^5^. At a shorter excitation wavelength of λ = 635 nm, the QF was limited by surface scattering and reached 4 × 10^7^.^30^

##### Self-Assembling

Self-assembling is one of the most efficient and cost-effective ways of fabricating all-polymer WGMRs. One example involves the fabrication of SU-8 microbottle WGMR lasers doped with various dyes, including LDS698, rhodamine B, rhodamine-6G (R6G), and rhodamine 123, which have demonstrated single-mode lasing at various colors (from green to red) and WGM performance with the QF of 10^5^ to 10^6^.^19^ Moreover, geometrically isotropic single (2–10) μm diameter microspheres (MSs) formed from self-assembled π-conjugated alternating copolymers displayed WGM photoemission. QFs of these MS WGMRs were as high as ∼100 for the 2 μm diameter MSs and ∼600 for 10 μm diameter MSs (Figure 1A–H). These relatively small QFs resulted from the inhibition of total internal reflection of the emission in the MS because of the MS’s large curvature.^10^ To compare, QF of ∼10^4^ was determined for single MS WGMR of self-assembled π-conjugated alternating poly­[(9,9-dioctylfluorene-2,7-diyl)-(5-octylthieno­[3,4-*c*]­pyrrole-4,6-dione-1,3-diyl). After laser irradiation, highly degenerate spherical modes split into multiple lines. That is associated with the geometrical distortion of the MS into the prolate spheroid, induced by optical excitation and photo-oxidation of the polymer.^33^ Finally, WGM performance was examined for homo- and heterotropic self-assemblies of π-conjugated alternating energy-donating and energy-accepting copolymer blend MSs. The efficient donor-to-acceptor Fürster resonance energy transfer (FRET) inside the MS and the blending ratio were confirmed by photoluminescence (PL), recording a systematic yellow-to-red color change. The QFs of the WGM PL lines ranged from 320 to 2200 (Figure 1D–H).^31^

**Figure 1:**
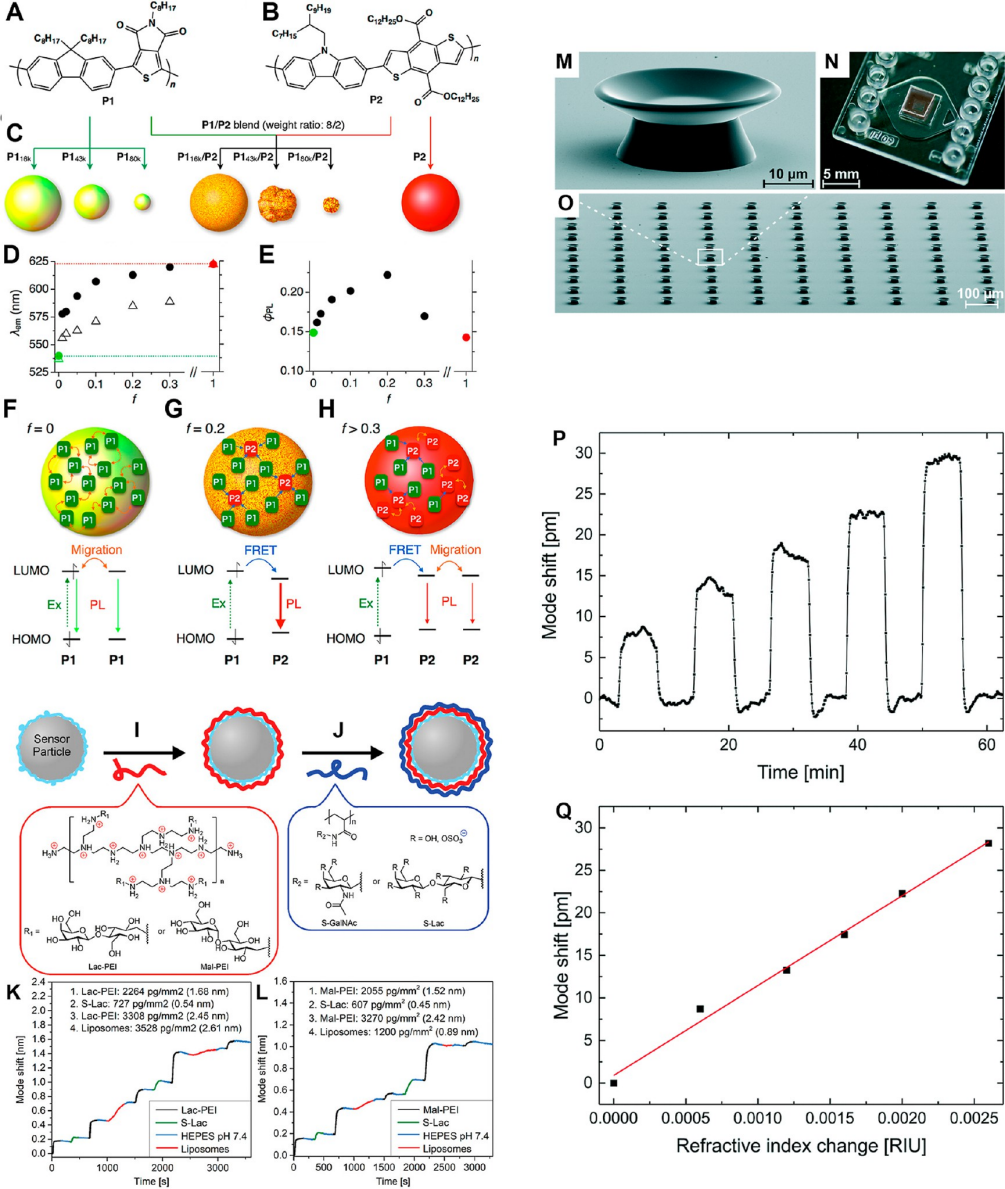
(A–H) A single-mode dye-doped SU-8 epoxy-based photoresist microbottle WGMR laser based on π-conjugated alternating dyed-doped SU-8. (A,B) Structural formulas of π-conjugated alternating copolymer microbeads of the size <10 μm. (C) Schematic structures from the copolymer blend self-assemblies. (D) Plots of the maximum wavelength of PL (λ_em_) and (E) PL quantum yield (ϕ_PL_) versus P1_16k_/P2 ratio (*f*) for films of the microspherical WGMRs. (F–H) Energy migration and energy transfer inside a single microspherical WGMR with (F) *f* = 0, (G) *f* = 0.2, and (H) *f* > 0.3. Adapted with permission from ref 31 Copyright 2016 American Chemical Society. (I–L) Liposome-embedded glycopolymer-based LbL multilayer thin film-coated WGMR. (I,J) Deposition of (I) cationic glycopolymers and (J) anionic glycopolymers on polystyrenesulfonate and poly­(allylamine hydrochloride) (PSS/(PAH/PSS) precoated WGM sensor particles via LbL self-assembly. (K,L) A shift of the WGM mode with time for LbL coating with liposomes (*d* ≈ 100 nm), S-Lac, and (K) Lac-PEI or (L) Mal-PEI in HEPES buffer (pH = 7.4). Adapted with permission from ref 14 Copyright 2022 Wiley. (M–Q) All-polymer PMMA WGM microgoblet lasers fabricated on a polysulfone substrate. (M) A single PMMA microgoblet laser supported on a polymer pedestal made from lift-off resist. (N) An array of 100 microgoblet lasers, fabricated by parallel solution-based processing, and (O) integrated into a sensing microfluidic chip. (P) Mode shift with time in the microgoblet laser mode is associated with an increase of the refractive index from 1.3335 to 1.3355, which is displayed upon injection of glycerol–water solutions. (Q) Mode shift against the RI change of 10.56 nm/RIU calculated from the linear fit (red) applied to the data. Adapted with permission from ref 15 Copyright 2015 Royal Society of Chemistry.

Multilayered and multicomponent WGMRs were fabricated on the basis of liposome-embedding glycopolymer-based layer-by-layer (LbL) self-assembly of thin films (Figure 1I–L). Specifically, cationic (branched polyethylenimine-based) glycopolymers and anionic (sulfate- and sialic acid–based) glycopolymers containing *N*-acetylgalactosamine, lactose, and maltose were deposited on 10 μm diameter polystyrenesulfonate (PSS) and poly­(allylamine hydrochloride) precoated WGMR sensor particles. Moreover, negatively charged ∼100 nm diameter liposomes were incorporated by LbL self-assembly between layers of these polyelectrolytes to produce an optical WGMR sensor equipped with a potential liposome-based drug delivery system (Figure 1I,J). The progress in the LbL self-assembly and the liposome immobilization was investigated, among others, by measuring WGM resonance wavelength shifts. Because only a 5 nm thick lipid layer of liposomes contributes to changes in RI, the WGM measurements revealed successful adsorption of an average 2.61 nm thick layer of liposomes on the lactose-polyethylenimine glycopolymer, while for the maltose-polyethylenimine glycopolymer, this layer was as thin as 0.89 nm (Figure 1K,L).^14^ By showing this subtle difference in a complex system, the researchers demonstrated that WGMR technology was sufficiently sensitive for investigating the content and the structure of multilayered and multicomponent biomedical composites.

##### 3D Microprinting

Moreover, 3D microprinting technology was used to fabricate all-polymer WGMRs. For example, arrays of SU-8 suspended-disk WGMRs (of radii of 230 and 160 μm) were fabricated using an optical 3D microprinting maskless stereolithography setup equipped with a high-power UV-light source and a digital micromirror device as a high-speed spatial light modulator. Coupling 230 and 160 μm radius WGMRs, operating in spectral ranges of 1500–1415 and 1520–1540 nm, with biconically tapered OFs resulted in the WGMRs’ QFs of 6.4 × 10^3^ and 4.9 × 10^3^, respectively.^34^

##### Two-Photon Polymerization

Two-photon polymerization (TPP) is a highly versatile direct laser writing technique that enables the fabrication of 3D micro- and nanostructures with submicron precision.^76,77^ TPP relies on the nonlinear absorption of tightly focused femtosecond (FS) laser pulses to induce localized polymerization within a photosensitive resin, thereby allowing accurate 3D structuring without LbL assembly. This technique is particularly appealing for the fabrication of polymer-based WGMRs, where the optical performance critically depends on structural precision, surface smoothness, and geometric control.^43–46^ TPP enables the production of resonators with finely tunable dimensions and excellent circular symmetry, which are essential for achieving high QFs.^43^ The QFs reported for polymer-based WGMRs fabricated via TPP generally range from 10^4^ to 10^5^, reflecting a balance between fabrication precision and material absorption limits,^44–46^ Moreover, TPP compatibility with a broad range of photopolymerizable materials, including transparent and low-loss polymers, makes it suitable for integrated photonic applications.^46^ Examples of materials commonly used for WGMRs fabrication via TPP include zirconium/silicon sol–gels^43,45^ and acrylic-based polymers.^44^ Importantly, TPP also facilitates monolithic integration of resonator structures onto prepatterned substrates, including optical fibers or waveguides, without the need for additional alignment or bonding steps.^78^ These capabilities make TPP a promising approach for the scalable fabrication of all-polymer WGMRs with application potential in sensing, optofluidics, and compact photonic systems.

##### Microfluidic Fabrication

All-polymer WGMRs were fabricated using microfluidics. For instance, a 2 mm rod-shaped WGMR composite of green light-emitting π-conjugated poly­[2,5-bis­(20,50-bis­(200-ethylhexyloxy)­phenyl)-*p*-phenylenevinylene] and a UV-transparent 1,4-cyclohexanedimethanol divinyl ether matrix was prepared by solution injection in a transparent plastic tubing. Once excited, the WGMR showed the 15-h underwater laser action in the range of 514–522 nm, detected by a fiber-coupled detector.^35^ Likewise, R6G-doped polydimethylsiloxane (PDMS)-based in-elastomer WGMR droplets were fabricated by a needle-dipping method. When solidified in the elastomer and excited, these easily deformable droplet microcavities of diameters of ∼60 to 90 μm displayed deformation red light-shifted WGM resonance wavelengths in the 570–610 nm range with the QF of ∼10^3^.^36^

Acrylamide-based MS WGMRs doped with quantum dots were fabricated using microfluidic flow-focusing droplet generators prepared via soft lithography in PDMS. The devices were molded from silicon wafers using SU-8 epoxy-based photoresist and bonded to glass substrates by oxygen plasma treatment. In the microfluidic chip, a water-in-oil emulsion system was used, where the aqueous dispersed phase contained acrylamide monomers, cross-linkers, initiators, and, optionally, quantum dots, while the continuous phase consisted of mineral oil with a nonionic surfactant and a polymerization catalyst. The droplet formation was pressure-controlled to ensure stable emulsification. Although the resulting MSs exhibited some size polydispersity (coefficient of variation, CV ≈ 20%), the method provided a robust route for producing optically active polymeric WGMRs with tunable composition. The fabricated MSs demonstrated QF of the order of 10^6^, confirming their suitability for high-sensitivity optical applications.^79^

Well-defined polymer printing was applied to enhance the WGM performance of dye-doped polymer droplets. For example, inkjet printing-based layer stacking was employed to fabricate in-spot hyperbranched TZ-001/TZ002 LDS798 dye-doped droplet microdisks of QF of ∼10^7^.^37^

Recently, an all-optical 940 nm (laser diode)-pumped polymer WGMR laser was prepared by pumping the photoluminescent poly­[(9,9-dioctylfluorenyl-2,7-diyl)-*alt*-*co*-(1,4-benzo-(2,10,3)­thiadiazole)] into a capillary tube of the wall thickness and inner diameter of 2.3 and 6 μm, respectively. The microfluidic all-polymer WGMR (QF of 5.17 × 10^3^) was devised by establishing an air-(capillary wall)-polymer structure with the RI of 1–1.45–1.55, respectively, thus fulfilling the RI scaling law. As a result, the all-polymer WGMR laser continuously emitted 2.8 s long light pulses of wavelength tunability over 13 nm. That corresponded to a tuning rate of 0.58 nm/(W cm^2^) and stability after 130 min under an irradiation power density of 17.32 W/cm^2^.^17^ Recently, a droplet-inspired pressure-modeled and temperature-insensitive MS WGMR was fabricated by forming a millimeter-scale UV-curable adhesive NOA65-based MS WGMR on a (superoleophobic material)-coated quartz slide. Composed as a droplet-like structure with an NOA65-core layer, this WGMR exhibited the maximal contact angle of 134° and the unique ability for under-pressure (pressure exerted by a force of 0–27 N) remodeling manifested by over 25% diameter variation without impacting the QF higher than 10^5^. Moreover, when pressurized, the sandwich-like structure allowed the tuning of the equivalent thermal expansion coefficient so that the temperature response range was dynamically adjusted from 0.12 to −0.056 nm/°C. Particularly, the temperature increase from 20 to 150 °C caused the contact angle of the NOA65 WGMR to decrease from 134 to 117°, whereas, for a similarly prepared PDMS droplet control, this drop was from 116 to 58°. In the 1549–1551 nm range, a redshift of WGM is observed in the temperature range of 25 to 32 °C; at the pressure generated by a force of 0 N, the shift equals ∼1 nm. Increasing pressure, exerted by a force of 0 to 27 N, and temperature from 26 to 32 °C result in the maximal WGM redshift drop from 1 nm to below −0.3 nm, thus turning the shift into blueshift. Moreover, under the pressure imposed by a force of 10 N, a temperature response of NOA65 WGMR was 0.006 nm/°C.^38^ According to the theory of elastic-optical effects, this value corresponds to pressure-induced RI changes of 10^–4^ to ∼10^–5^, which provides higher temperature stability than standard silicon WGMRs.

#### Applications of All-Polymer WGMRs

##### Sensing Applications of All-Polymer WGMRs

All-polymer WGMRs are devised for the lab-in-a-droplet sensing of humidity, temperature, vapors, liquids, and biomolecular compounds. For example, an ultrasmooth surface self-assembly SU-8 WGMR with QF of ∼10^4^, packaged in a cured PDMS support, was devised for microlensing and ultrasensitive (120 pm/°C) temperature sensing.^39^ Similarly, PMMA-based pyrromethene 597-doped microgoblet laser (QF of ∼10^5^), fabricated by spin-coating, mask-based lithography, wet etching, and thermal reflow, and integrated into a microfluidic chip, enabled refractometric examination of glycerol–water solutions, demonstrating a bulk RI sensitivity of 10.56 nm/RIU (Figure 1M–Q).^15^ Likewise, an array of 14.74, 74.44, and 165 μm PMMA MS WGMRs allowed optical temperature sensing upon excitation with a 635 nm laser. In the range of 25–35.8 °C, the stability of the array of 18 74.44 μm MS WGMRs was the highest, and their sensitivity was 0.001 nm/K.^22^

##### Gas and Vapor Sensing Applications of All-Polymer WGMRs

All-polymer WGMRs serve as sensors of gases and vapors. Recently, the (gas binding)-induced swelling of pillared SU-8 microdisk WGMR (QF of 5 × 10^4^) allowed the determination of pentanoic acid vapors with the highest sensitivity (23 pm/ppm) and the LOD of 0.6 ppm.^26^ Moreover, π-conjugated poly­(9,9-dioctylfluorene-*alt*-benzothiadiazole)-based MS WGM microlaser (size-dependent QF of 0.5–1.5 × 10^3^), fabricated by emulsion-solvent evaporation, determined acetone vapor with a sensitivity of 0.21 nm/ppm with the LOD of 90 ppb.^40^ Furthermore, a trichromatic single-mode laser microresonator, MR (QF of ∼10^5^) with directional far-field emission, based on R6G-doped polymer droplet self-assembled in 3D-curved microcavities, was devised for sensitive acetic acid vapor determination with the sensitivity of 186.5 pm/min and the LOD of 5 min (Figure 2A–F).^11^ Similarly, 5 μm PS MS WGMRs (QF of 1080) doped with (β-cyano)-appended oligo­(*p*-phenylenevinylene) were prepared by the miniemulsification method to detect vapor compounds, including alcohols, ketones, furan, and aromatic hydrocarbons, namely, benzene, toluene, and xylenes. By penetrating the PS matrix, those compounds effectively interact by affinity with the matrix to cause WGM resonance wavelength shifts. Those shifts increase in the order of MeOH < acetone < THF < EtOH < methyl ethyl ketone < *p*-xylene < benzene < *m*-xylene < *o*-xylene < toluene, with a sensitivity of 1.49 nm/μL.^41^

**Figure 2:**
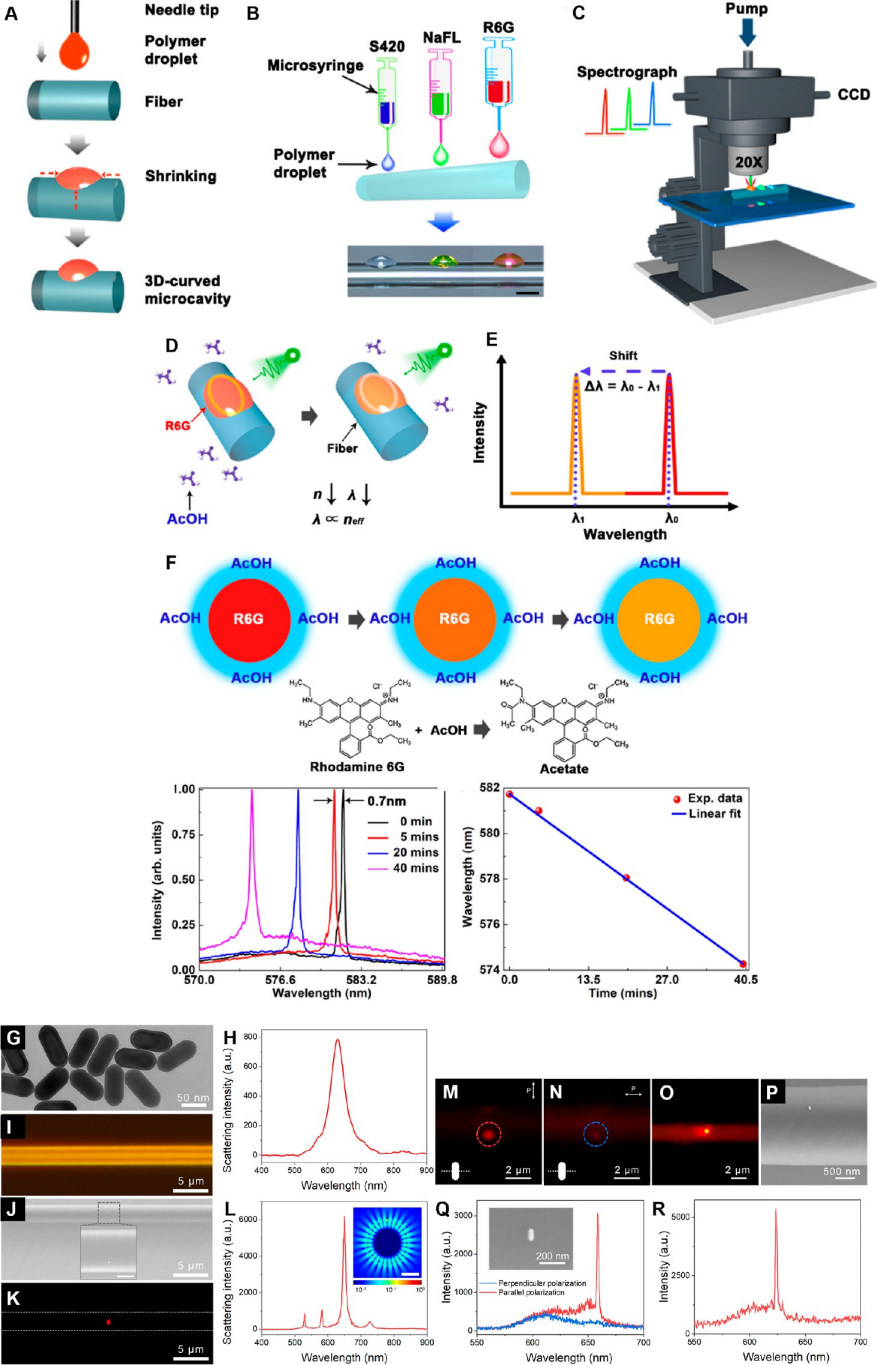
(A–F) A drop-based trichromatic self-assembly 3D-curved microcavity single-mode laser for acetic acid vapor sensing. (A) (Self-assembly)-based fabrication, (B) Schematics and optical microscopy images of trichromatic 3D-curved microcavities (scale bar represents 80 μm), (C) experimental setup for excitation and signal collection, (D,E) scheme and principle of the acetic acid vapor sensing method, respectively, and (F) reaction between the R6G fiber device and the acetic acid vapor, emission characteristics at different reaction times, and wavelength shift as a function of the reaction time. Adapted with permission from ref 27 Copyright 2021 American Chemical Society. (G–R) Gold nanorod-coupled WGM microfiber displaying hybrid photon-plasmon lasing. (G–L) Strong mode coupling enables bandwidth narrowing. (G,H) TEM imaging and scattering intensity measurements for gold nanorods, respectively. (I) Bright-field optical microscopy image of PMMA WGMR microcavity, (J) SEM, and (K,L) dark-field scattering images and spectra of a gold nanorod-coupled PMMA microfiber (*d* = 2.5 μm). (M–R) Polarization-sensitive lasing. (M,N) Optical microscopy images of an active 2.5 μm thick microfiber taken at parallel and perpendicular polarization, respectively (*P* = 3.59 MW/cm^2^). (O,P) Optical microscopy and SEM image, respectively, of an active 2.0 μm thick microfiber (*P* = 3.59 MW/cm^2^) and (Q,R) corresponding polarization-sensitive lasing spectra and spectrum, respectively, of the lasing emission. Adapted with permission from ref 49 Copyright 2022 American Association for the Advancement of Science.

##### Applications of All-Polymer WGMRs for Sensing of Biorelevant Analytes

Finally, a SU-8-based microfluidic label-free WGMR sensor for glucose was fabricated by hybrid FS laser micromachining. A sensing activity of SU-8 WGMR (QF of 5 × 10^3^), incorporated in a glass monolithic lab-on-a-chip (LOC), was reported by using the biotin–streptavidin binding assessment, in which the sensor’s sensitivity was 61 nm/RIU, and the LOD was 0.0048 RIU.^42^

##### Real-Time Monitoring of Polymer Transformations

Polymer-based WGMRs are increasingly being leveraged for a range of emerging applications that extend well beyond classical refractometric sensing. One notable area is the real-time monitoring of physical and chemical transformations in polymer materials. For instance, WGM tracking was used to study solvent diffusion in glassy polymer MSs, enabling label-free monitoring of swelling, glass–rubber transitions, and surface dissolution in materials like PMMA and polystyrene.^80^ These phenomena were quantitatively described using a perturbative model, linking RI gradients and resonator morphology to observable resonance shifts.

### Polymer Optical Fiber (POF) Resonators and Lasers

Another group of polymeric WGMRs involves polymer optical fiber (POF) lasers. The current section discusses the design and implementation of POF WGMRs, which exhibit significant potential for optical sensing and laser applications. Due to the unique characteristics of polymer waveguides, POF-based WGMRs offer versatility in various photonic systems.

#### Fabrication Methods of POF WGMRs

##### Direct Drawing

An exemplary POF WGM low-threshold microlaser of QF of ∼6 × 10^3^ was fabricated, based on R6G-doped (10–100 μm) thick PMMA microfibers, using a direct drawing method.^42^ The lasing emission polarization was modulated between the transverse electric (TE) and transverse magnetic (TM) modes by adjusting the orientation of the pumping laser. Specifically, TM-mode emission was achieved if the electric field of the pumping laser was aligned parallel to the POF axis, while TE-mode emission occured if the field was perpendicular.^42^ As such, POF WGMs are applicable in liquid crystal (LC) displays, modulators, and optoisolators.^42^ Recently, a waveguide-coupled WGM polymer microring resonator was fabricated directly onto the planarized surface of a D-shaped single-mode optical fiber, serving simultaneously as both the substrate and light-guiding platform. The flat region of the fiber, prepared via FS laser ablation and selective etching above the fiber core, enabled efficient in-plane light coupling without the need for external optical alignment. FS laser-induced TPP defined the polymer resonator, ensuring high spatial resolution and integration fidelity. The use of angled sidewalls in the D-fiber geometry, combined with a tapered waveguide design, significantly improved the mode-matching efficiency between the waveguide and the resonator, increasing power transmission from 0.215 to 0.835 while suppressing backscattering losses from 0.025 to 7 × 10^–6^. The QF of the resulting MR was 4.86 × 10^3^ at 1508.14 nm, demonstrating the feasibility of monolithically integrated polymer-based WGM devices within optical fiber platforms.^58^

##### Electrospinning

POFs are usually fabricated by common electrospinning or direct drawing from a solution. Initially, metal-based (organic dye)-doped POF WGM lasers were fabricated by incorporating monodisperse (10 μm) long and (60 nm) thick Ag nanowires into the gain medium of R6G fluorophore. Precisely, using a preform drawing method, a low-threshold WGM lasing of high photostability was prepared from an (Ag nanowire)-incorporating R6G-doped PMMA OF laser. The QF of genuine R6G-POF WGMRs was 10^3^, whereas the QFs of Ag-R6G-POF WGMRs exceeded 10^4^. Apparently, the presence of the Ag nanowires in R6G-POF WGMRs stabilized the laser action and enhanced the rate of radiative decay of the R6G active medium, thus providing low pump pulse energy for exciting WGM modes in the microcavity.^47^ In another study, encapsulating 10.43 μm PS MSs into Ge-doped silica core single-mode microstructured OFs suspended within three hollow channels was employed to produce a “Mercedes-shaped” in-capillary WGMR. This POF WGMR features two launch/collection schemes: core input/scattering output and sphere input/core output. The latter enabled exciting the MS WGMRs externally to POF with QF ≈ 2.2 × 10^3^.^48^

##### Plasmon-Enhanced POF WGM Microcavity Fabrication

Likewise, a low threshold (∼2.7 MW/cm^2^) single-mode lasing with a bandwidth narrower than 2 nm was obtained using a (Rhodamine 101)-doped (∼2.5 μm) diameter PMMA OF WGMR microcavities coupled with a single plasmonic Au nanorod with the average diameter and length of 38 and 84 nm, respectively (Figure 2G–R).^49^ By attaching the Au nanorod perpendicularly to the OF long axis, one can generate a dominant hybrid photon-plasmon mode and enhanced coherence because of the coupling between the localized surface plasmon resonance mode of the Au nanorod and WGM of the OF (Figures 2G and 3L). Besides, the oriented placement of the nanorod on the WGMR surface allows a polarization-sensitive lasing behavior. The output lasing intensity is maximal if the polarization of the polarizer is parallel to the long axis of the nanorod. In contrast, if the polarization of the polarizer is rotated to the direction perpendicular to the long axis of the nanorod, the output intensity is minimal (Figure 2M–R).^49^

**Figure 3:**
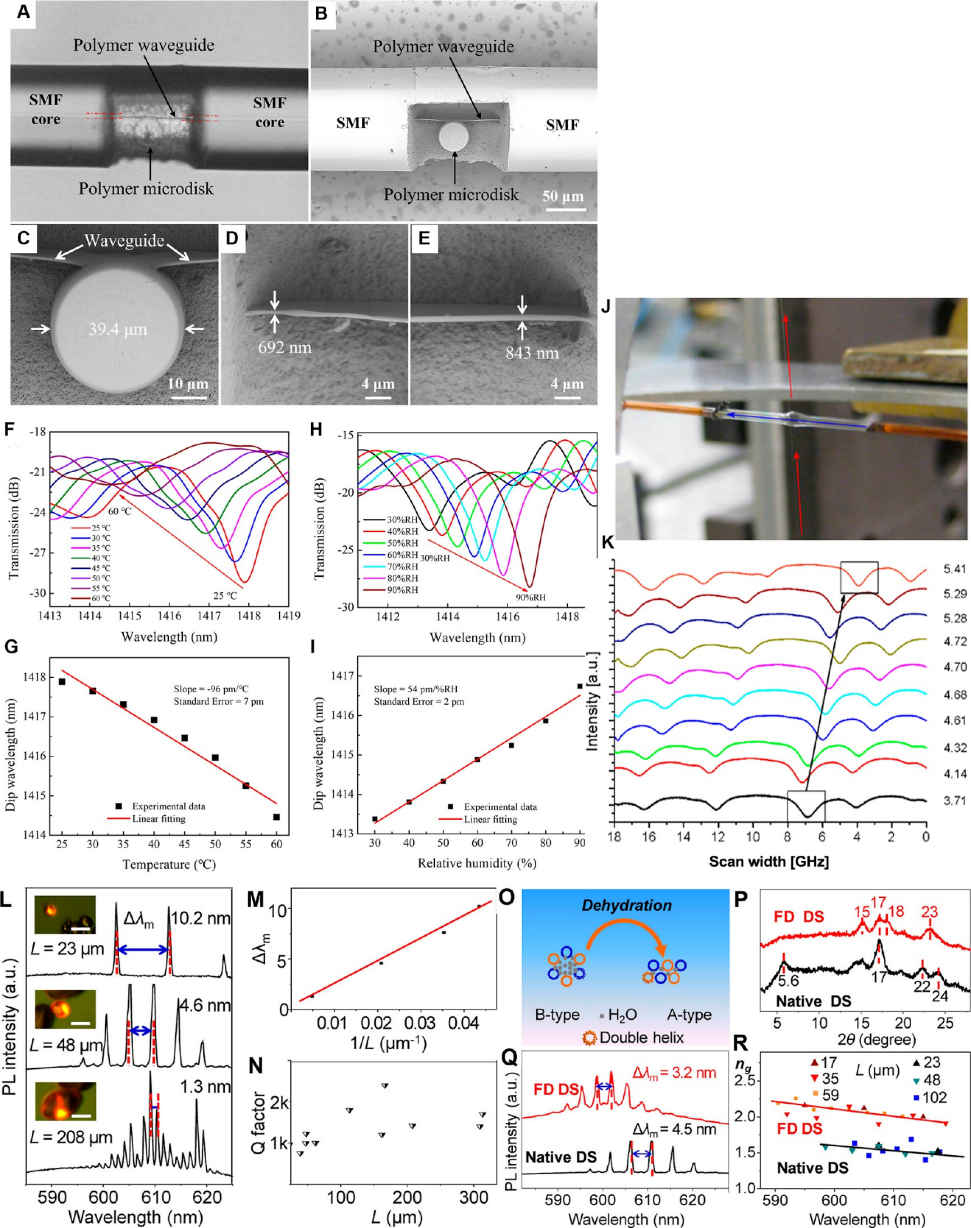
(A–I) Temperature and humidity sensing by in-fiber polymer microdisk WGMRs prepared by femtosecond laser micromachining of single-mode optical fiber. (A) Optical microscopic and (B) SEM images of the (C) microdisk and (D) waveguide on the left side and (E) right side. (F,G) 25–60 °C temperature-dependent spectral evolution of in-fiber microdisk WGM resonances and (G) linear fit of the dip wavelength versus temperature. (H,I) 30–90% humidity-dependent spectral evolution of in-fiber microdisk WGM resonances and (I) linear fit of the dip wavelength versus ambient humidity characteristics. Adapted with permission from ref 11 Copyright 2021 American Chemical Society. (J,K) pH sensing using WGMR of a silica hollow bottle. (J) Experimental settings showing a relative position of the fiber with respect to the resonator; vertical and horizontal arrows show directions of light propagation in the tapered fiber and the flow direction of buffer solution introduced into a hollow bottle resonator, respectively. (K) WGM frequency shift with the pH increase in a polymer film-coated bottle resonator in an external sensing configuration. The arrow indicates the shift of the boxed WGM, perturbed by temperature fluctuations. Adapted with permission from ref 66 Copyright 2019 Elsevier. (L–R) Starch-based biomicrolasers. (L) PL spectra and images (scale bar of 20 μm) of the (cation dye)-doped, 4-[*p*-(dimethylamino)­styryl]-1-methylpyridinium (DASP^+^), starch granules with three different sizes. (M) Relationship between Δλ_m_ and 1/(L, μm) of the dye@starch (DS) granules. (N) Experimental hot QF versus cavity length (L, m) of different DS granules. (O) Blue-shifted lasing wavelength resulting from dehydration-induced B-type to A-type structural transformation of potato DS granules. (P) X-ray diffraction patterns of native B-type potato starch and the starch after freeze-drying (FD) treatment. (Q) Lasing spectra of a single granule before and after FD treatment. (R) Plots of the group RI versus the wavelength of native DS and FD DS granules with different sizes. Adapted with permission from ref 24 Copyright 2017 American Chemical Society.

##### Multifiber Microstructures and Dynamic Coupling

Finally, because of the high processability and functional versatility of polymers, POFs are used as flexible and tunable materials for constructing adjustable multifiber microstructures. A recent study demonstrated quasi-3D coupled WGMR microcavities consisting of two intersected self-assembly POFs based on disodium 4,4′-bis­(2-sulfonatostyryl)­biphenyl, poly­(vinyl alcohol), and cetylmethylammonium bromide. The coupling strength between two POFs, of diameters of 21.8 and 24.5 μm and FSRs of 1.85 and 1.64 nm, respectively, was dynamically micromanipulated by adjusting the coupling angle (0–90°) and the coupling distance (0–450 nm). This design was employed to prepare the single-mode lasing from multiple modes. The single-mode lasing of the highest output intensity (pump fluence of 115.2 μJ/cm^2^, the emission wavelength of ∼442 nm) was derived as a synergistic effect obtained from the POFs microcavity composed of POFs placed in parallel (angle of 0°), attached one to another (distance ∼0 nm).^50^

#### Applications of POF WGMRs

##### Temperature, Humidity, and Vapor Sensing Applications of POF WGMRs

Because of their sensing properties, POF WGMRs are applicable to determining relative humidity (RH) and temperature. For example, a hollow-core fiber internally integrated polymer microdisk WGMR, composed of a nanoscale thick (128 μm) long polymer waveguide and a (39.4 μm) diameter polymer microdisk, printed by FS laser-induced TPP, was used to detect temperature and RH changes in a WGMR RI-dependent manner (Figure 3A–K). Within this all-silica-fiber polymer WGMR, the polymer components were placed in close proximity and were finely integrated within a single-mode fiber, thus providing a resonator cavity with a sufficiently high evanescent field to transmit optical signals (Figure 3A–E). The smooth surface of this laser-polymerized WGMR resulted in a fine QF of 2.3 × 10^3^ at the wavelength of 1416.6 nm and RI of 1.543, calculated numerically. Because of the distinguishable thermo-optic and thermal expansion of the swellable polymer WGMR, manifested in RI changes and WGM resonance wavelength shifts, this in-fiber WGMR enabled sensitive measurement of temperature and RH. Temperature changes were recorded in the linear range of 26–60 °C at 55% RH, with a sensitivity of −95 pm/°C, and RH in the linear range of 30–90%, with a sensitivity of 54 pm/% RH (Figure 3F–K).^11^ Furthermore, a waveguide-coupled polymer WGM microring resonator, directly integrated into the planar surface of a D-shaped single-mode, demonstrated temperature sensing capability with a sensitivity of −193 pm/°C. This compact, monolithically integrated resonator exhibited a QF of 4.86 × 10^3^ at 1508.14 nm and offered a robust, fiber-based platform for future biochemical and environmental sensing applications.^58^

A B-type starch-based POF microlaser was devised by interhelical inclusion of 4-[*p*-(dimethylamino)­styryl]-1-methylpyridinium as an active medium into potato starch granules (Figure 3L–R). The quasi-linear amylose and highly branched amylopectin chains of the starch form an ellipsoidal structure, thus serving as WGMR. The low-threshold microlasing action can be obtained by doping this microellipsoid granule resonator (major axis of 67 μm and minor axis of 47 μm) with the laser dye and excitation with a 400 nm FS pulse laser (Figure 3L–N). Because this biolaser was sensitive to the structural transformation of the starch from B-type (hydrated state) to A-type (dehydrated state), manifested by a blue-shifted lasing wavelength following WGMR freeze-drying (FD) treatment (Figure 3Q,R), it could efficiently serve as a humidity biosensor.^24^

##### Biosensing Applications of POF WGMRs

Moreover, a microstructured WGM PS MS-attached POF resonator was devised for dynamic biomedical self-referenced sensing of neutravidin in undiluted immunoglobulin-deprived human serum, using a biotin-neutravidin model (Figure 4A–H).^51^ The sensor was constructed so that one 15 μm MS WGMR acted as a dynamic reference to compensate nonspecific binding events as well as environmental RI and temperature changes, whereas the other MS WGMR, of virtually identical size, RI sensitivity, and surface area, was used for detecting neutravidin (Figure 4A,B). For 600 s, neutravidin diluted in the serum in four concentrations of 25, 50, 100, and 400 nM was detected by biotinylated MS WGMRs that exhibited a steady increase in surface density beyond the initial WGM wavelength shift because of the high RI of the serum. The detection followed Langmuir adsorption of neutravidin onto the biotinylated WGMR surface. However, at a concentration as low as 5 nM, neutravidin remained undetectable (Figure 4C–H).^51^

**Figure 4:**
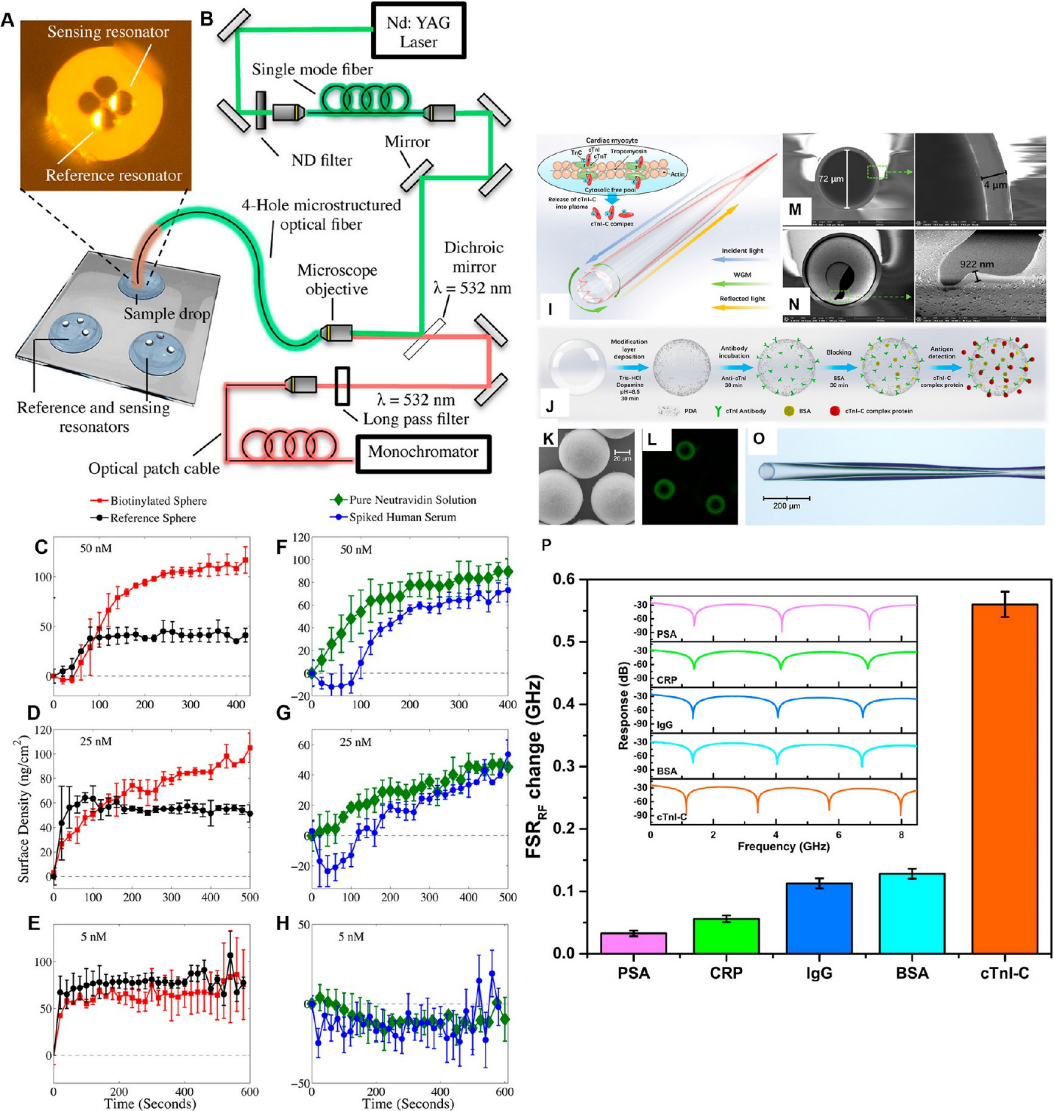
(A–H) Self-referenced biosensing of neutravidin with a microstructured optical fiber coupled with dye-doped polystyrene MS WGMRs. (A,B) Scheme of a bright-field microscopy setup of two 15 μm diameter MS WGMRs positioned onto the tip of a four-hole microstructured optical fiber. The setup includes a neutral density (ND) filter to control light intensity and a neodymium-doped yttrium aluminum garnet (Nd/YAG) laser for excitation. (C–E) Individual MS WGM responses with the time of the biotinylated (red trace) and reference (black trace) MS WGMR responses after dipping into human serum samples spiked with (C) 50, (D) 25, and (E) 5 nM neutravidin. (F–H) The corrected binding kinetics of the sensor in the spiked human serum samples (blue trace) and binding kinetics in the pure neutravidin solution (green trace). Adapted with permission from ref 51 Copyright 2016 American Chemical Society. (I–P) Detection of the cardiac troponin I–C (cTnI-C) complex—a marker of myocardial damage—with a fiber-integrated WGM optofluidic chip enhanced by a microwave photonic analyzer. (I) Schemes of the myocardial sarcomere with the cTnI–C complex released after myocardial damage and (J) the surface functionalization and detection using an optofluidic polydopamine-embedding HGMS WGMRs. (K) SEM and (L) fluorescence microscopy image of the HGMS-immobilized FITC-(cTnI-C) antibody. SEM images of (M) the etched capillary with a wall 4 μm thick and (N) the broken HGMS with a 922 nm thick wall. (O) Optical microscopy image of the WGM fiber probe embedded with HGMS. (P) Radiofrequency (RF) spectra and radiofrequency free spectrum range (FSR_RF_) changes of the cTnI-C and other nonspecific samples for selectivity evaluation using prostate-specific antigen (PSA), C-reactive protein (CRP), immunoglobulin G (IgG), and bovine serum albumin (BSA), each at 1 ng/mL in 0.01 M PBS (7.2 < pH < 7.4). Adapted with permission from ref 81 Copyright 2022 Elsevier.

##### Optofluidic Devices and Lab-on-a-Chip (LOC) Applications of POF WGMRs

Because of miniaturized dimensions, flexibility, processability, and exceptional photostability, POF WGMRs are successfully applicable for devising optofluidic LOCs and sensors. In optofluidic devices, light is used to control the flow of fluids at a micrometric scale or to manipulate light with on-chip fluidic processes. For example, simple dip-pen nanolithography on a 3D prefabricated on-chip goblet-shaped passive PMMA WGMR was employed to obtain an optofluidic biosensor for streptavidin. For that, the WGMRs were coated with multifunctional light-guiding fluorophore-labeled phospholipid inks. Streptavidin was biosensed by capturing it in a 3D mobile lipid layer. By diffusing into deeper layers of this biosensor, the streptavidin molecule interacted with the biotin molecule head groups, thus intercalating. That resulted in the WGM resonance wavelength shift. The adhesion of 50 nanomoles of streptavidin for 270 s caused a temporal red shift of ∼126 pm, whereas the adhesion of nonspecific bovine serum albumin (BSA) caused only a ∼14 pm shift.^52^ Continuously, WGMR fabrication was employed to construct, e.g., thin-walled microfabricated optofluidic ring resonators (μOFRRs) and microcapillary-based optical microbubble resonators (OMBRs), enabling sensitive detection of RI changes within the microcavity or surrounding fluids. The first technological trials employed a cylindrical OFRR exploiting the Vernier effect. It allowed measuring RI in an aqueous solution with a sensitivity of 2510 nm/RIU and the QF of 355 that corresponded to the LOD of 1.6 × 10^–5^ RIU.^53^

A more advanced strategy involved the micromachining of a monolithic on-chip μOFRR sensor for volatile organic compounds (VOCs). It consisted of a (∼300 nm) thick PDMS film-coated, (250 μm) thick SiO_*x*_ μOFRR cylinder with a quasi-toroidal mode-confinement section, a microfluidic interconnection channel, a capillary insertion port, and an OF probe alignment feature supported on an Si chip. Measuring reversible WGM resonance wavelength shifts caused by 50-fold RI changes resulting from the (vapor partition)-induced swelling of the PDMS film in the cylinder, this WGM μOFRR, displaying QF of 1.15 × 10^4^ measured at λ = 1550 nm, allowed the determination of ppm traces of benzene, toluene, ethylbenzene, *m*-xylene, and *n*-octane, with the sensitivity below 1 pm/(mg/m^3^).^54^ Analogously, VOCs were detected using a μOFRR sensor combined with a Si-microfabricated 2D gas chromatographic microsystem (μGC) devised as a detector. The PDMS film-lined μOFRR chip consisted of a hollow-contoured SiO_*x*_ cylinder connected with a photodetector with an OF and coupled with a 1550 nm tunable laser. The WGM resonance wavelength shifts were generated within the chip wall by the transient sorption of VOC vapors eluting into the PDMS film. In the second step, the VOC vapors were isothermally separated using the μGC. In the 7-VOC mixture matrix, 1,4-dioxane, toluene, 4-methyl-2-pentanone, *n*-octane, ethylbenzene, 3-heptanone, and *n*-nonane were determined with the LODs of 15, 8, 12, 7, 11, 19, and 16 ng, respectively.^55^

Biologically relevant compounds were sensed using WGM μOFRRs. In a recent study, UV lithography-based 3D printing was employed to fabricate an optofluidic sensor for (horseradish peroxidase-streptavidin)-based enzyme-linked immunosorbent assay (ELISA) of vascular endothelial growth factor (VEGF). By using microlasing of this PDMS/SU-8 WGM μOFRRs of diameters of 116, 146, and 195 μm, resulting in QFs of 9800, 7000, and 5800, respectively, this sensor determined the VEGF analyte with the LOD of 17.8 fg/mL, which, advantageously, is 2 orders of magnitude lower than that of commercial kits.^56^ Finally, cardiac troponin (cTnI-C), a biomarker of myocardial damage, was sensed using a polydopamine-functionalized LC molecules-modified hollow glass microsphere (HGMS)-embedded capillary-fiber probe containing immobilized FITC-cTnI-C antibody, and incorporated in a reflective WGM μOFRR microlasing immunosensor (Figure 4I–P). Using a PDMS optofluidic chip, integrated with a time-delay-dispersion-based microwave photonic analyzer, cTnI-C was determined by exploring the dispersive delay difference between the sensing laser and the reference laser in OF (Figure 4I–O). Converting cTnI-C binding-induced slight wavelength shifts of the sensing laser into a radio frequency response enabled successful cTnI-C determination in liquids with the LOD of 0.59 ng/mL and resolution of 1.2 fg/mL (Figure 4P).^17^

Following the same concept, cTnI-C was sensed by an OF immunosensor exploiting a prefabricated LC {dimethyloctadecyl­[3-(trimethoxysilyl)­propyl]­ammonium chloride}-containing HGMS. In this OF, HGMS served as both a reservoir entity and a sensing element. Upon functionalization with LCs, the QF of the unmodified HGMS WMGR dropped from 1.94 × 10^4^ to 2.63 × 10^3^. The analyte was determined both directly by using an FITC-conjugated cardiac antibody attached vertically to the HGMS surface and indirectly by utilizing the birefringence of LCs, i.e., the dependence of the LC RI on the polarization and propagation direction of light. Because of the vertical orientation of the LC molecules against the HGMS surface, cTnI-C binding by the antibody disturbs the orientation of the LC layer, thus altering the effective RI of the functionalized HGMS manifested as a WGM resonance wavelength shift. Upon binding cTnI-C, the effective RI of LCs changed from extraordinary RI (*n* = 1.67) to ordinary (*n* = 1.51). cTnI-C was determined in the concentration range of 0–40 ng/mL by measuring FSRs of (non-LC)-modified and LC-modified HGMS WGMRs. For (non-LC)-modified HGMS WGMR immunosensor, the addition of 10, 20, 30, and 40 ng/mL cTnI-C caused no significant changes in FSR (FSR of 7.09 and 7.04 for 0 and 40 ng/mL cTnI-C), whereas, for LC-modified HGMS, the FSR values were as high as 5.56, 6.67, 6.78, 7.02, and 7.22 for 0, 10, 20, 30, and 40 ng/mL cTnI-C. The LOD was 1.103 ng/mL, and the validation assessment revealed that during 27 min, 1 ng/mL cTnI-C caused a 0.37 nm WGM resonance wavelength shift.^57^

### (Polymer Shell)-(Inorganic Core) Composite WGMRs

(Polymer shell)-(inorganic core) WGMR composites consist of inorganic cores (typically silica) coated with organic polymer shells. Notable examples include silica MR cores combined with metal–organic frameworks (MOFs) (Figure 5A–O),^71^ single-walled carbon nanotubes (SWCNTs) (Figure 5P–S),^72^ or molecularly imprinted polymer (MIP) shells.^73^ However, SWCNTs and MIPs are not typical for these composites but rather specific examples of advanced designs. Most polymer WGMR composites are based on silica, which acts as the scaffold for the MR. This category includes both silica dioxide supports and silica nanoparticles (SiNPs). The operational mechanism of some core–shell MS WGMR composites relies on the electrostriction effect, where (external electric field)-induced surface and body forces cause elastic deformation in the MSs. However, this effect is typical of certain materials and conditions rather than being a general feature of all those composites. These elastic morphological deformations of the polymer shell and (or) Si core are detectable by laser light propagating through the OF and around the MS, resulting in the WGM resonance wavelength shifts, thus causing so-called “morphology-dependent resonances.” Initially, polymeric multilayer dielectric Si-core MS WGMRs were fabricated based on (i) single-PDMS-layer spheres, (ii) multilayered PDMS cores coated with BaTiO_3_ and PDMS films, and (iii) Si cores coated with thin layers of uncured PDMS-based coatings. All these WGMRs provided a QF of 10^6^ and sensitivity of 1.7 pm/(kV/m) for PDMS MS, 2.5 pm/(kV/m) for PDMS-based triple layer MS, and 0.2 pm/(V/m) for silica/PDMS MS. The sensitivity of the PDMS film-coated Si-core MS was the highest because of the presence of the soft, yield stress-liquid outer layer of PDMS.^61^

**Figure 5:**
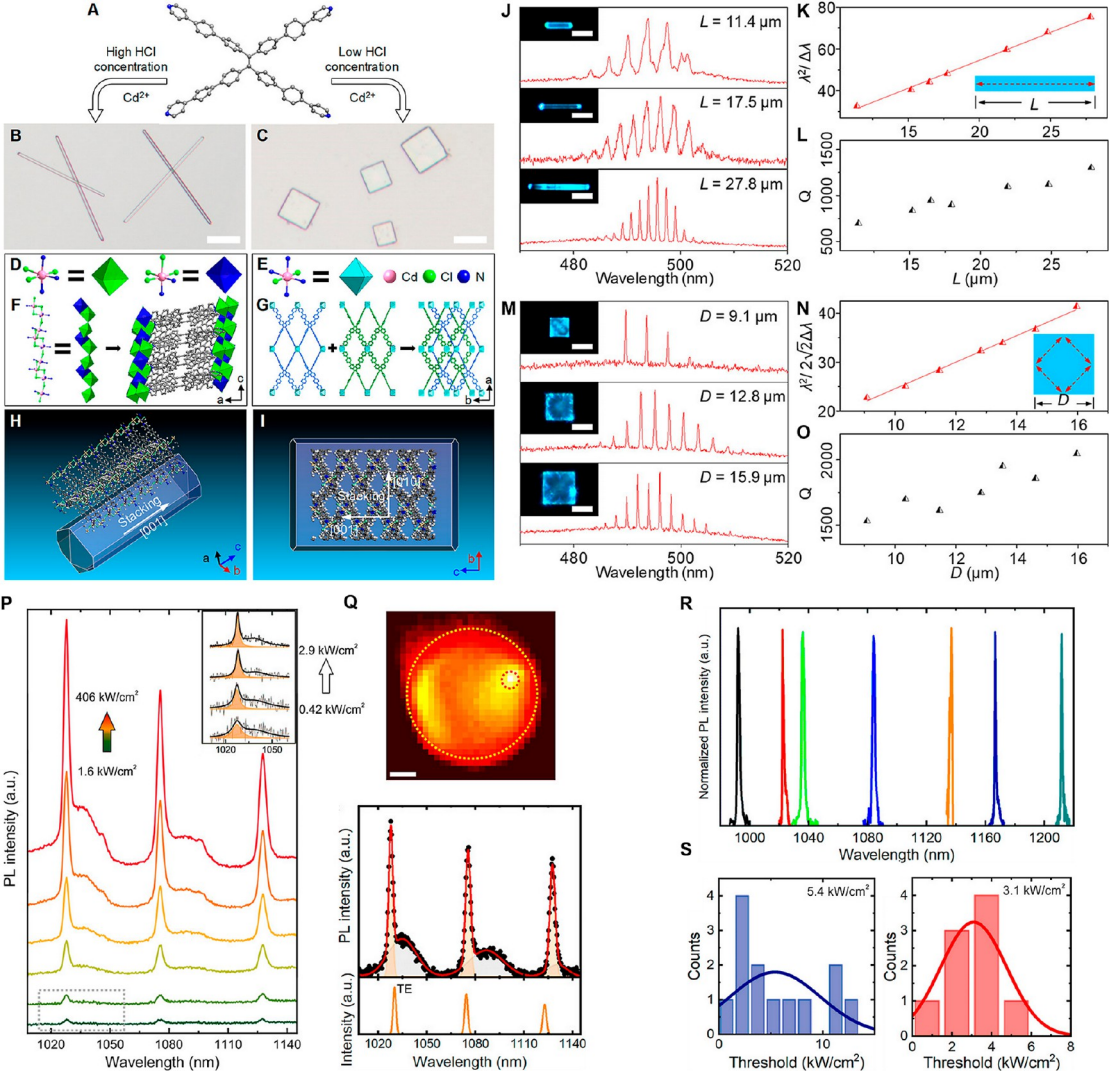
(A–O) Metal–organic framework (MOF)-based WGM microlasers. (A) The structure of 1,1,2,2-tetrakis­(4′-(pyridine-4-yl)-[1,1′-biphenyl]-4-yl)-ethene monomer of the MOF. (B,C) Bright-field microscopy images of the MOF microwires and microplates (scale bars indicate 10 μm). (D,E) Molecular structures of Cd^2+^-containing nodes in the MOF microwires and microplates. (F,G) Molecular structures of the Cd–Cl–Cd chains in MOF microwires and microplates. (H,I) A theoretically predicted morphology of the MOF crystals. (J,M) PL spectra of the MOF microwires and microplates with different sizes (scale bars indicate 10 μm). (K) The λ^2^/2Δλ dependence on *L* for the MOF microwire. (N) The λ^2^/2^1/2^Δλ dependence on *D* for the MOF microplate. (L) A plot of the QF against *L*. (O) A plot of the QF against *L*. Adapted with permission from ref 71 Copyright 2020 American Chemical Society. (P–S) Room-temperature lasing from semiconducting chemically functionalized SWCNTs coupled to a polystyrene MS. (P) Pump power-dependent PL spectra. (Q) Wide-field PL of a dopant-based SWCNT nanolaser (scale bar indicates 1 μm) with a PL spectrum and simulated TE resonance modes supported by a 5.4 μm diameter polystyrene MS. (R) Emission spectra of SWCNT-based nanolasers spanning a wide wavelength range and (S) histograms of the lasing thresholds of the steady and (excited state)-based nanolasers. Adapted with permission from ref 72 Copyright 2022 American Chemical Society.

#### Fabrication Methods of (Polymer Shell)-(Inorganic Core) Composite WGMRs

##### Silica-Based Core–Shell Composites

Continuously, a micrometer-sized composite of SiO_2_ microparticle cores coated with a conjugated poly­(1-vinylpyrrolidone-*co*-vinyl) shell, prepared via seeded Knoevenagel dispersion polymerization, revealed polymer shell thickness-dependent WGM lasing properties. The thicker the polymer shell, the lower the lasing threshold and the broader the emission spectral range.^62^ The WGM performance of (polymer shell)-(silica core) composite resonators can be enhanced by adding spacers that create a nonfouling interface and reduce nonspecific adsorption onto the resonating core without compromising sensitivity, thus increasing the signal-to-noise ratio and accuracy.

A straightforward wet-processing approach was recently introduced^5^ for fabricating free-standing WGMRs composed of π-conjugated organic molecules and spherical silica gel MSs. The method involves the physical blending of luminescent π-conjugated fluorophores, including 4,4′-bis­(2-butyloctyloxy)-*p*-terphenyl (BPT), with spherical silica MSs in ethanol, followed by slow solvent evaporation to enable self-assembly. This facile route yields MRs that do not require sophisticated lithography or surface functionalization and allows for fabrication via physical entrapment rather than covalent modification.^5^

Polymer-functionalized silica WGMRs are exploited as OMBRs. For instance, ellipsoidal 320 μm OMBRs, fabricated using two reverse discharges focused on silica microcapillaries, were internally coated with polyhexamethylene biguanide layers via filling and sintering. A comparison of QF of uncoated and polymer film-coated OMBRs showed the QF values of 1.08 × 10^5^ and 7.33 × 10^4^, respectively.^65^

Integrating polymer films into ultrahigh-quality (QF > 10^7^) silica optical WGMR cavities enhanced the MRs’ optical properties. In particular, silica microtoroids were fabricated using lithography and CO_2_ laser reflow, which were then spin-coated with PMMA and PS and subsequently thermally reflowed to produce smooth surfaces. The overlap of the optical field distribution with the silica–polymer–air was modeled using finite element method (FEM) simulations, which allowed calculation of the material-limited QF and direct comparison with the experiments.

The PMMA and PS film-coated silica microtoroids exhibited material-loss-limited QFs, confirmed both experimentally and theoretically, with QFs > 10^7^ obtained for the microtoroids coated with the polymer films of the thicknesses below 80 nm. Besides, if the major (minor) diameter of the toroid were set to 40 (8) μm, then the percentages of the optical field in the polymer film, calculated as functions of the PMMA and PS film thicknesses spanning from 20 to 500 nm, increased exponentially, whereby the percent of the optical field in the PS coating film was greater. That was because of the higher contrast of refractive indices between silica and PS compared to silica and PMMA.^60^

In a similar study, a scalable, nondestructive replica molding resulted in the fabrication of high-quality polymer-based microtoroid arrays, exploiting a Vicast optical polymer. In detail, the PDMS and Vicast film-coated silica WGMR replicas were cast by curing silanized molds at 75 °C. The diameter of the obtained WGMRs was 45 μm, the FSR values were consistent with theoretical predictions of 11.5 nm, and intrinsic QFs for Vicast and PDMS film-coated WGMRs were up to 5 × 10^6^ and 2 × 10^6^, respectively, with minimized thermal and scattering effects. The PDMS and Vicast material losses were measured at 1319 and 1550 nm using a Metricon system, namely, a prism coupling the measurement setup with a system of planar waveguides.

The Metricon PDMS absorption values indicated that bulk absorption limited the measured loss and not the waveguide scattering. For Vicast, the Metricon-derived data yielded material-limited QFs of 2.71 × 10^6^ at 1319 nm and 3.11 × 10^6^ at 1550 nm, which appeared consistent with the measured intrinsic QFs.^59^

##### Metal–Organic Frameworks (MOFs) and Nanocomposites

Another group of (organic shell)-(inorganic core) polymer WGMR composites involves transition metal-containing materials. For instance, a hexagonal ZnO microrod incorporated in the interface of the PMMA matrix and integrated with a p­(+)-GaN semiconducting substrate was used to fabricate a heterostructured microlaser diode (Figure 6A–J).^67^ Likewise, a self-rolled-up oxide tubular WGM microcavity coated with poly­(acrylic acid)/polyethylenimine films designed in a polymer/oxide/oxide architecture was devised. The silica-supported nanocomposite consisted of an Al_2_O_3_/Y_2_O_3_/ZrO_2_/Al_2_O_3_ film of a 30/12/24/30 nm thickness, respectively, and a superficial 33.2 nm polymer film (Figure 6A–G).

**Figure 6:**
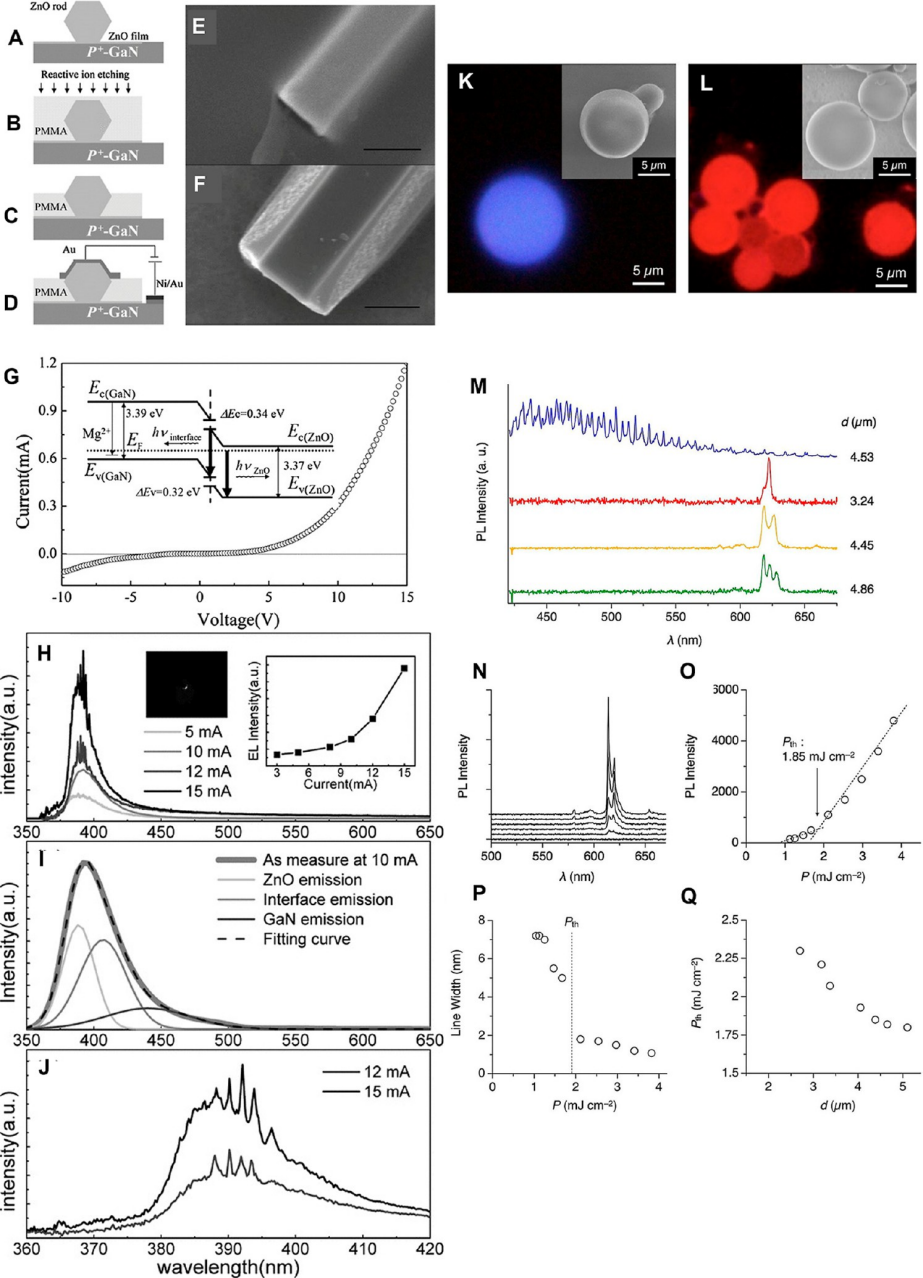
(A–J) ZnO-microrod/*p*-GaN heterostructured WGM microlaser diodes. (A–D) Steps of fabrication of ZnO microrod/GaN heterojunction diodes. (E) SEM image of the ZnO microrod coated with a PMMA thin film by spin-coating before and (F) after reactive ion etching. (G) The current–voltage curve for the ZnO microrod/GaN heterojunction diodes with a schematic of the band diagram. (H–J) The electroluminescence spectra and Gaussian fits from the ZnO microrod/GaN heterojunction diodes for currents of 3 to 15 mA applied. Adapted with permission from ref 67 Copyright 2011 Wiley. (K–Q) Energy transfer-assisted WGM lasing in π-conjugated polymer/Eu hybrid MS WGMRs. (K,L) Fluorescent micrographs and SEM images of MS WGMRs from poly­[(9,9′-dioctyl-9*H*-fluoren-2,7-yl)-5,5′-(2,2′:6′,2″-terpyridine; a monomer; (K,L) the polymer–Eu complex, which this monomer forms with Eu­(III) thenoyltrifluoroacetonate trihydrate, [Eu­(tta)_3_] × 3H_2_O. (M–Q) PL intensity spectra of the MS WMGRs: (M) PL intensity spectra of a single MS WGMR of the monomer (blue, MS diameter, *d* = 4.53 μm) and the polymer–Eu complex with *d* = 3.24 (red), 4.45 (orange), and 4.86 μm (green); (N) PL intensity spectra of a single MS WGMR of the polymer–Eu complex with *d* of 4.38 μm with *P* of 1.04, 1.25, 1.67, 2.11, 2.97, and 3.82 mJ/cm^2^ (from bottom to top). (O,P) Plots of the PL intensity at 615 nm and the bandwidth of the PL versus optical pump power (*P*), respectively. (Q) The plot of the lasing threshold (*P*_th_) versus *d*. Adapted with permission from ref 70 Copyright 2018 Wiley.

A 0.7Pb­(Mg_1/3_Nb_2/3_)­O_3_–0.3PbTiO_3_ piezoelectric crystal was combined with a poly­[9,9-dioctylfluorenyl-2,7-diyl] microring cavity to obtain an electrotunable WGM microlaser. It was devised by employing inkjet printing to prepare piezoelectric crystals with an ultrahigh piezoelectric strain constant of ∼3000 pm/V, providing continuous dynamic modulation under an external electric field. The photoluminescent 53, 67, and 85 μm diameter poly­[9,9-dioctylfluorenyl-2,7-diyl] microrings were inkjet-printed onto PDMS-layered piezoelectric crystals.^69^

The 3.2 μm diameter copolymer MS WGMRs were prepared using the vapor diffusion and miniemulsion methods.^70^ Noticeably, lanthanides display remarkable magnetic properties that can be used to manufacture polymer WGMR composites. For example, a WGMR magnetometer operating in the hertz-to-kilohertz frequency range was fabricated by integrating a microtoroid WGMR with an Nd micromagnet glued to the film of the supporting polymer.^70^

Recently, the coordination-mode-tailored fabrication of the single-crystalline 1D microwire and 2D microplate MOF microlasers was attributed to strong optical confinement and low-threshold microcrystal lasing. Remarkably, the Cd^2+^-noded MOFs containing 1,1,2,2-tetrakis­(4′-(pyridin-4-yl)-[1,1′-biphenyl]-4-yl)-ethene ligands were synthesized using hydrochloric acid of various concentrations to modulate both the MOFs nucleation and the morphology and optical features of the microlasers. Diamagnetic Cd^2+^ ions were used to avoid the quenching of fluorescence (Figure 5A–I).^82^ Recently, dye-doped SWCNTs self-assembled on PS MS WGMRs were employed to fabricate near-infrared semiconducting nanolasers of the estimated QF of (3.5–4) × 10^3^ (Figure 5P,Q).^72^

(Polymer shell)-(inorganic core) composite WGMRs were fabricated on the basis of MIPs. MIPs, the so-called biomimetic receptors and catalysts, also known as “plastic antibodies”, “artificial receptors,” and “semisynthetic enzymes”, are synthetic, target-compound dedicated, nanostructured materials. They are fabricated via polymerization of functional and cross-linking monomers in the presence of a target analyte, which is often initially used as the template. Upon template extraction from the resulting MIP, this MIP becomes a matrix of target-selective molecular cavities of the size-, shape-, and affinity-controlled recognition sites complementary to the binding sites of the target. Concerning MIP-based WGMRs or microlasers, in a recent study, (100–200) μm tapered single-mode OF-derived MS WMGRs were coated with films of inorganic silane-based FITC-selective MIPs, prepared by sol–gel transition, onto either silica-on-silicon wafers or onto silica MSs, via manual or automated dip coating. Two methods of FITC extraction were compared, i.e., chemical extraction with the ethanol-chloroform-acetic acid and ethanol-acetonitrile-acetic acid solution, and oxygen-plasma extraction. The average QF of uncoated MS WMGRs was 1.241 × 10^7^. Coating the MS WMGRs with FITC-MIP decreased the QF to 1.243 × 10^6^.^73^

Finally, to mention bioengineering silica WGMRs with natural biopolymers, including nucleic acids, peptides, and polysaccharides, an evanescent wave-excitable DNA-coated microtoroidal sensor was devised to validate a highly sensitive, real-time detection of DNA hybridization, i.e., a crucial process exploited in genetics diagnostics. Precisely, the silica WGMRs were fabricated using photolithography, then etching, and then CO_2_ laser reflow, creating microtoroids with QF of 2.2 × 10^7^. Next, 20-nucleotide ssDNA, labeled with 6-carboxyfluorescein (6-FAM) of λ_excitation_ = 495 nm and λ_emission_ = 517 nm, was deposited on silica toroidal microcavities. Those were presilanized with (3-glycidyloxypropyl)­trimethoxysilane to immobilize ssDNA using an epoxy approach. During the real-time determination, a solution of the complementary ssDNA, labeled with cyanine 5 (Cy5) of λ_excitation_ = 635 nm and λ_emission_ = 650–670 nm, was injected and hybridized to the sensor’s surface. The Cy5-ssDNA fluorescence was excited via the evanescent wave of a silica microcavity and monitored using a tapered OF-coupled spectrograph.

The biosensor performance allowed for the discrimination between the specific DNA hybridization and nonspecific interactions using temporal fluorescence analysis, with a high signal-to-noise ratio and the LOD for ssDNA ranging from 1 nM to 2 μM. However, the LOD was moderate because of light scattering and water absorption.^74^

The WGMRs were fabricated from fused silica MSs coupled to a tapered optical fiber for excitation.^83^ These WGMRs were housed in a PDMS-based liquid chamber filled with aqueous glycerol solution at a precisely controlled temperature. Temperature cycling was implemented using a thermoelectric cooler (TEC) with active feedback control from a high-precision thermistor with ±0.01 K resolution, enabling adiabatic and hysteresis-free measurements. Unlike previous coating-based stabilization techniques, this method does not require precise microfabrication or surface patterning, and it maintains biocompatibility, making it suitable for biomolecular sensing applications.

#### Applications of (Polymer Shell)-(Inorganic Core) Composite WGMRs

##### Environmental Sensing Applications of (Polymer Shell)-(Inorganic Core) WGMRs

WGMRs coated with polymer films are very promising for gas-sensing applications. For instance, an OMBR coated with a polyhexamethylene biguanide film was employed to measure CO_2_ concentrations. The sensor sensitivity and LOD were 0.46 pm/ppm and 50 ppm, respectively, in the linear dynamic concentration range of 200 to 700 ppm of CO_2_. The selectivity of the sensor was verified by testing it against other gases, including nitrogen, hydrogen, and argon, thus demonstrating its high selectivity for CO_2_.^84^

Optical frequency-shift refractometric pH sensing was demonstrated for swellable pH-sensitive hydrogel-embedded *N*-isopropylacrylamide particles layered on the inner surfaces of silica-hollow-bottle WGMRs (Figure 4P).^66^ The pH-responsiveness of this WGMR, with the acquired resolution of 0.06 pH, was evaluated by measuring mode frequency shift as a function of the pH of the buffer. That was monitored by the throughput of the tunable diode laser coupled into the WGMR via a tapered fiber (Figure 4P). Correlating the sigmoid-shaped mode shift-pH titration curves with turbidity studies enabled the association of pH increases with decreases in the RI of the polymer particles and frequency shifts of internal evanescent components of WGM resonances (Figure 4P).^66^

Moreover, WGMR composites have been used for humidity sensing. For example, a self-rolled-up oxide tubular WGM microcavity coated with poly­(acrylic acid)/polyethylenimine films was devised to determine environmental RH.^31^ The Modus operandi of this sensor relied on the polymer swelling upon water vapor sorption, which thickened the WGMR walls and red-shifted the WGM resonance wavelength (Figure 2U–W). As a result, the polymer WGMR composite facilitated the humidity determination better than a genuine MR by displaying a sensitivity of 130 pm per relative humidity unit.^31^

##### Optoelectronic Applications of (Polymer Shell)-(Inorganic Core) WGMRs

(Polymer shell)-(inorganic core) composite WGMRs also play a key role in optoelectronic applications, including microlaser diodes and tunable lasers. The ZnO-PMMA-GaN heterostructured microlaser diode mentioned above illustrates the potential for creating electrically driven WGM microlasers with applications in compact optical circuits or sensing devices. This diode exhibits electrically driven WGM lasing (QF of 550) and μ-PL in the range of 350–500 nm. Compared to the optical pumping, a relatively low QF results from the light leakage at the GaN/ZnO and the PMMA/ZnO interfaces.^67^

WGMRs composed of π-conjugated organic molecules and spherical silica gel MSs exhibited reproducible high-quality WGM lasing owing to the spherical morphology of the silica gel. These MRs could be readily transferred onto various substrates without structural deformation. Their optical performance strongly depended on the silica-to-fluorophore ratio, which determined both the lasing threshold and spectral purity. This feature makes the method a promising and accessible alternative for producing WGM-active MSs for photonic applications.^5^

Similarly, the electrotunable microlasers fabricated from piezoelectric crystals offer precise wavelength control under an external electric field, which is essential for applications requiring real-time tuning of optical properties, including optical communications and dynamic sensing systems. For pump fluence values 16.8, 19.0, and 13.3 μJ/cm^2^, the QF values were 3280, 3530, and 4620, respectively. The electrostrain-induced properties of these WGMR microlasers were evaluated by measuring wavelength shifts in the 0–0.5 kV/mm direct current (dc) electric field range. The wavelength was tuned to ∼0.7 nm by applying a dc electric field of 0.48 kV/mm.^69^

Organic–inorganic polymer WGMRs and lasers were constructed from MOFs (Figure 5A–O). MOFs have become attractive novel materials for miniaturized lasers because they combine the excellent stability of inorganic semiconducting materials with the processability and ability to self-assemble organic materials. As a result, the prepared MOF lasers feature a typical shape-dependent microcavity effect, i.e., 1D microwires act as Fabry–Perot MRs, whereas 2D microplates act as WGM MRs. Regarding these microplate WGMRs, particularly, they exhibit PL in the spectral range of 480–520 nm, express relatively high RI, and their QFs are of the order of ∼10^3^ (Figure 5J–O), which should be considered as high for novel MOF MRs.^71^

Polymer WGMR composites exploit exquisite multicolor luminescence of lanthanides based on up-conversion and energy transfer between lanthanide ions (Figure 6K–Q).^70^ For example, energy transfer-assisted WGM lasing was obtained by coupling the Eu^3+^ luminescence with WGMs in self-assembled π-conjugated poly­[(9,9′-dioctyl-9*H*-fluoren-2,7-yl)-5,5′-(2,2′:6′,2″-terpyridine) alternating copolymer (Figure 6K,L). Once laser-excited, in the absence of Eu^3+^, the copolymer MSs revealed WGM PL in the broad spectral range of 420–680 nm. In the presence of Eu^3+^, however, upon excitation with a laser, the copolymer acted as an energy transfer donor, manifested as a sharp WGM Eu^3+^-characteristic PL in a narrow spectral range of 615–630 nm (Figure 6M–Q), thus demonstrating efficient photoinduced energy transfer to Eu^3+^.^70^

Moreover, polymer MSs were coupled with SWCNTs to enhance the WGM resonance (Figure 5P–S).^72^ This resonance was enhanced because of the diameter-tunable electronic structures that provide the efficient spatial overlap between the gain material and the photonic microcavity modes. This overlap allows for the generation of stimulated emissions in the system and excitonic lasing from the semiconducting SWCNTs. Besides, low lasing thresholds are obtained thanks to the 4-nitrobenzene diazonium tetrafluoroborate and (4-iodoaniline)-induced sp^3^ hybridized dopants. The estimated average lasing thresholds for the pristine E11 SWCNTs-PS-WGMRs and dopant-functionalized E*11 state-based SWCNTs-PS-WGMRs were 5.4 and 3.1 kW/cm^2^, respectively (Figure 5R,S). Here, E11 denotes the fundamental excitonic transition in pristine semiconducting SWCNTs, while E*11 corresponds to defect-induced states resulting from chemical functionalization. These results suggest facile tunability of the WGMR laser, which is expected to be applicable in near-infrared optoelectronics.^72^

##### All-Optical Switching Applications of (Polymer Shell)-(Inorganic Core) WGMRs

More recently, attention has shifted toward dynamically reconfigurable WGMR systems enabled by switchable and stimuli-responsive polymers. For instance, WGMRs incorporating phase-transition hydrogels, including poly­(*N*-isopropylacrylamide) (PNIPAM), exhibit thermally driven mode switching and resonance tuning due to reversible volume changes. Building on this concept, single-mode lasing in hydrogel-filled capillary WGMRs with tunable emission characteristics was demonstrated.^85^ This lasing behavior was controlled by temperature across the hydrogel’s transition point, showcasing the potential of phase-change polymers for optical modulation and switching. Another significant study reported WGM microcavities functionalized with azobenzene-containing polymer layers exhibiting light-induced, reversible RI changes.^86^ In this way, functionalized materials have enabled all-optical frequency tuning and switching without requiring thermal or electrical actuation. This development is crucial for unlocking low-power, tunable, and programmable photonic devices based on polymer WGMR platforms, by combining the advantages of inorganic cores with the flexibility and functionality of polymer shells.

##### Biosensor Applications of (Polymer Shell)-(Inorganic Core) WGMRs

Recently, a thin-walled fused silica capillary WGM OMBR was used for the ultrasensitive label-free determination of cTnI-C (Figure 7).^49^ In this setup, the OMBR of diameter and wall thickness of ∼256 and ∼1.7 μm, respectively, was silanized using 3-glycidoxypropyltrimethoxysilan, internally functionalized with cTnI-C antibody and blocked with BSA, and then coupled into an OF (Figures 6C and 7A). The QF, sensitivity, and LOD in PBS (pH = 7.4) of the resulting biosensor were ∼1.5 × 10^5^, 6.3 nm/RIU, and 0.4 ag/mL, respectively. The biosensor was selective for cTnI-C against the interferents of human immunoglobulin and PSA (Figure 7D–G). These sensing properties make WGM OMBRs promising medical tools dedicated to the diagnosis of acute myocardial infarction treatment, being far below the clinical cutoff values of routine diagnostic devices used for myocardial damage.^49^

**Figure 7:**
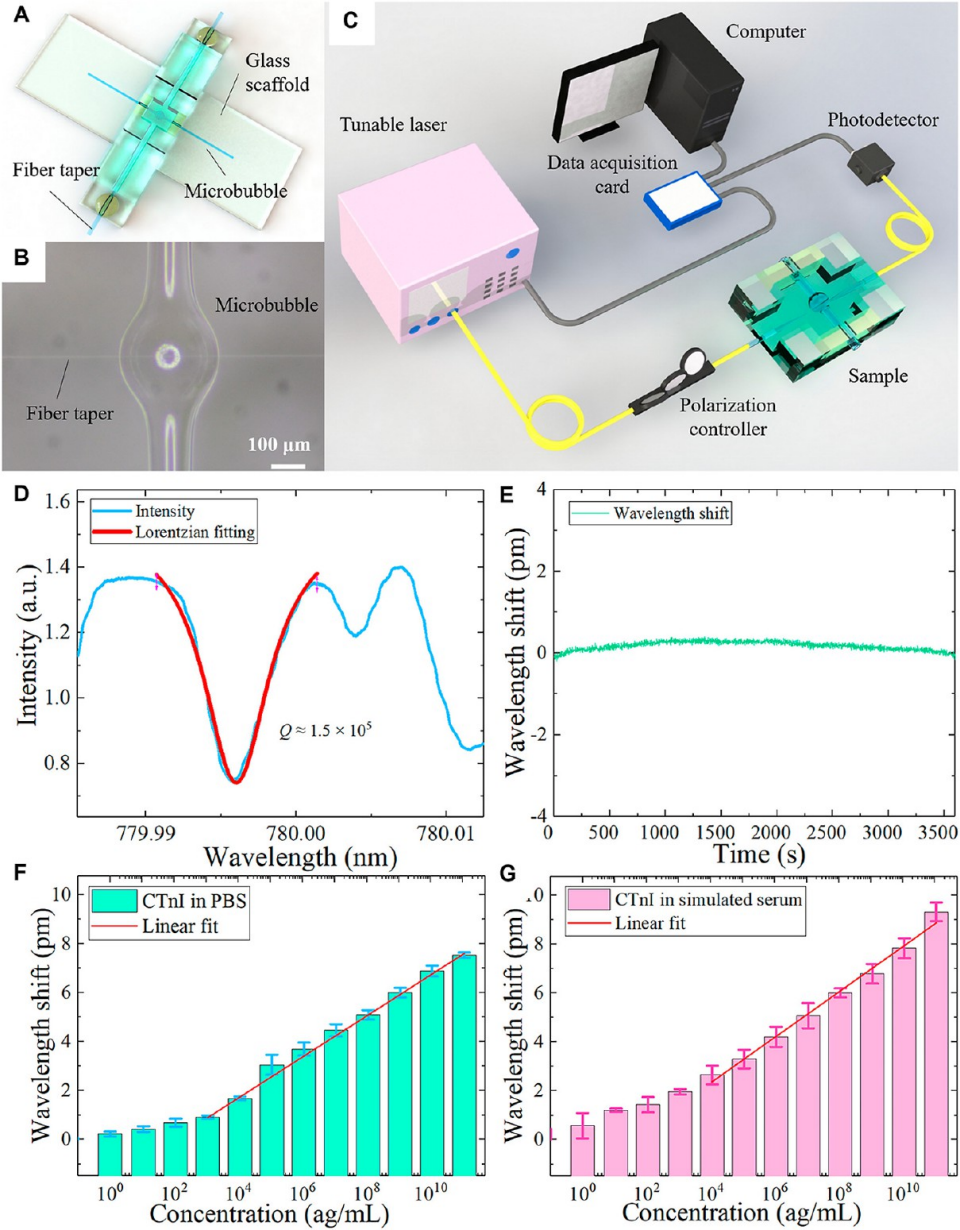
(A–G) Thin-walled microbubble WGMRs for label-free determination of cTnC-1. (A) A setup scheme, (B) a photograph of the microbubble and the fiber taper, and (C) an optical setup for label-free cTnI-C determination using packaged thin-walled microbubble WGMRs. (D,E) Optimization of the response of (D) the spectrum and (E) the time-dependent wavelength shift. (F,G) cTnI-C concentration-dependent resonance wavelength shifts in (F) PBS and (G) simulated serum. Adapted with permission from ref 49 Copyright 2022 Wiley.

For instance, silica WGMR surfaces with covalently bound fluorescein isothiocyanate-labeled PEG spacers appeared resistant to nonspecific adsorption of proteins, which allowed for devising well-tuned WGM microcavities.^63^ Likewise, using the avidin–biotin analyte-recognition-unit system, nonspecific adsorption of the lysozyme and fibrinogen interferents on the WGMR surface was significantly lower in the case of non-PEG film-coated SiO_2_ WGMRs, compared to the relevant control. The device responded with a 20–30 pm wavelength shift if exposed to 100–1000 μg/mL avidin.^64^

##### Thermal Nanosensing of Biomolecular Film Applications of (Polymer Shell)-(Inorganic Core) WGMRs

A hybrid WGM sensor was devised based on silica MSs (inorganic cores) immersed in aqueous glycerol solutions, enabling precise thermal stabilization of resonance wavelength shifts.^83^ This approach compensates for temperature-induced drifts by jointly tuning the MS radius and the thermal RI coefficient of the surrounding medium. Proper adjustment of the glycerol concentration in the aqueous environment enabled a 60-fold reduction in thermal sensitivity, reaching values as low as 0.15 pm/K for 62 μm silica MSs, compared to bare resonators in air. The thermally stabilized WGM platform allowed for high-resolution thermal characterization of adsorbed protein or (bio)­polymer films, which would otherwise be obscured by thermal noise. Specifically, the temperature-dependent optical responses of three representative macromolecular compounds, including dextran and poly­(diallyldimethylammonium chloride) (polyDADMAC), were investigated. The system enabled measuring their thermal RI coefficient and, for BSA, the molecular polarizability changes. The study demonstrated the potential of (polymer shell)-(inorganic core) WGMRs for label-free, temperature-resolved sensing of soft matter and biomolecular films, with relevance to biophysics, biosensing, and materials characterization.

##### Magnetometer Applications of (Polymer Shell)-(Inorganic Core) WGMRs

The robust WGMR composite was devised using silica microtoroids of major diameters of 120–150 μm and minor diameters of ∼10 μm, displaying the intrinsic QF of ∼5 × 10^7^. When packaged into a ∼200–500 μm film of the UV curable polymer, the microtoroid QF decreased to ∼10^6^ because of pressure-induced deformation of the encapsulating polymer. That was measured as a WGM resonance wavelength shift caused by a change in the polymer’s RI.

This subtle change was detectable by measuring the frequency of the magnetic field of the (500–2000) μm Nd magnets incorporated in the composite. The sensitivity of this magnetometer was as high as 880 pT/Hz^1/2^ at 200 Hz with a four-decade linear dynamic concentration range.^18^

##### Photonic/Optical Enhancement Applications of (Polymer Shell)-(Inorganic Core) WGMRs

Finally, in addition to WGMRs composed of (organic polymer)-(inorganic core) composites, an example of all-inorganic WGMRs based on silica polymeric films doped with transition metals is worth mentioning. Those were devised to exhibit enhanced or novel optical features. In particular, (107–109) μm diameter silica toroidal WGMRs, coated with Zr-doped silica sol–gel films, improved the performance of Raman-Kerr frequency combs by keeping the low dispersion in MRs while maintaining high-efficiency four-wave mixing. The Zr doping with 5, 10, and 15 mol % notably improved the optical performance, as indicated by the increase in Raman efficiency from 0.027 to 0.414%, while the Raman threshold decreased significantly from 4.19 to 0.82 mW for WGMR doped with 15 mol % Zr. The frequency comb span broadened from 150 to over 300 nm, which was beyond the optical spectrum analyzer LODs. This frequency comb span broadening occurred because of the combination of improvement of the Stokes and anti-Stokes Raman scattering, causing cascaded four-wave mixing (FWM) peaks from both Raman emission peaks, as well as the improvement in dispersion. In contrast to the significant improvement of Raman-Kerr effects displayed by the Zr-doped silica sol–gel film-coated toroids, the WGMRs QFs decreased from 1.34 × 10^8^ to 1.52 × 10^7^ because of material absorption losses.^75^

## Discussion

WGMRs have garnered significant attention in optical sensing applications mainly due to their high sensitivity and compact size.^3,87^ Traditionally, the WGMRs have been fabricated using inorganic materials, e.g., fused silica or inorganic crystalline materials.^88–92^ However, recent advancements have focused on utilizing polymers in various forms to enhance WGMRs’ performance and versatility. The present review article critically explores three emerging types of WGMRs, namely, all-polymer WGMRs, polymer film-coated optical-fiber WGMRs, and (polymer shell)-(inorganic core) composite-functionalized WGMRs. It evaluates the advantages of using polymers in WGM applications, discusses novelties introduced by these designs, and outlines future directions focusing on sensitivity and LODs.

Polymer-based WGMRs represent a breakthrough in photonics owing to their unique combination of high QFs, versatility in fabrication, and enhanced optical properties. These features make them invaluable for a wide range of applications in advanced optics and bioengineering. The current review delves into the exquisite properties of polymer WGMRs and explores their applications. The QF is a measure of a resonator’s ability to confine light within its structure. A high QF indicates low energy loss and prolonged light circulation, features essential for precision sensing and high-performance of optical devices. Polymer WGMRs have achieved significant advancements in QF, particularly in compact resonator designs. For instance, enhancing the QF in small WGMR may be achieved by optimizing the material and structural parameters in miniaturized dimensions.^65^ This innovation holds promise for applications, including biosensing, where compact, high-sensitivity devices are required.

Moreover, polymer-based WGMRs benefit from advanced fabrication techniques that enable rapid production and geometric versatility. Contemporary technologies, including 3D microprinting, allow for the devising of complex and customizable MR geometries, which are not feasible with traditional materials, including silica.^34,56^ This adaptability enhances the integration of polymer WGMRs into diverse photonic and optoelectronic systems. As showcased,^34^ the rapid production of high-precision structures has paved the way for scalable manufacturing of polymer-based photonic devices. This capability is particularly advantageous for prototyping and iterative designing in research and industrial applications.

Furthermore, polymer WGMRs exhibit intrinsic nonlinear optical properties, which can further be tuned through doping or structural modification.^93^ This feature is highly suitable for nonlinear optical processes, including second-harmonic generation and frequency mixing, which are crucial for applications in optical signal processing and wavelength conversion. For example, this applicability was explored using advanced organic non-polymer and polymer MRs, which allow for efficient light manipulation, thus expanding the functionality of photonic devices.^18^

The inherent material flexibility and diversity of polymer WGMRs enable the devising of tunable optical materials, which are essential for adaptive photonic systems. The thermo-optic effect in polymers, for example, allows precise control over resonance wavelengths through temperature modulation.^94,95^ This tunability is critical for applications in dynamic wavelength filters and tunable lasers. Specifically, single-mode lasing in polymer bottle MRs can be applied to construct highly precise temperature-tunable WGM lasers.^18^ These advancements facilitate the development of compact and adaptable light sources for optical communication and sensing technologies. Although protocols of surface functionalization, particularly via silanization, of WGMRs made from inorganic materials, including silica, are well established and extensively documented, numerous studies suggest that polymer-based resonators can also be effectively employed in biosensing applications.^42,56^ Those resonators can be used in biosensors to determine biocompounds with high sensitivity, leveraging their ability to monitor resonance shifts caused by changes in the surrounding environment. Moreover, their ease of integration into soft and flexible substrates enables the invention of wearable and implantable biomedical devices.

The biocompatibility of polymers is a critical characteristic that significantly broadens the applicability of WGMRs, especially in biosensing, where direct contact with biological samples is required. All-polymer WGMRs leverage the inherent biocompatibility of many polymeric materials, enabling their use in biological environments without adverse reactions. This feature allows devising sensors suitable for various biomedical determinations, including those of streptavidin,^52^ glucose,^42^ cTnI-C,^17,49,57^ and VEGF,^56^ as demonstrated with SU-8,^42,56^ PMMA,^52^ and PDMS-based resonators, respectively.^56^ PMMA, for instance, has been widely utilized in microgoblet lasers for refractometric sensing of glycerol–water solutions, highlighting its stability in aqueous environments relevant to biological applications.^15^ Similarly, PDMS is a frequently employed polymer in μOFRRs due to its excellent biocompatibility and flexibility, crucial for on-chip sample treatment and integration with microfluidic systems.^54–56^ Moreover, an epoxy-based SU-8 photoresist has successfully been integrated into microfluidic label-free biosensors, indicating its suitability for biomedical applications.^42,56^ PS MSs, while used for vapor sensing applications,^41^ also find utility in biosensing if integrated with OFs for self-referenced determination of biomolecular compounds, including neutravidin in human serum, harnessing PS’s established use in biological assays.^51^ Furthermore, advancements in hydrogels, e.g., PNIPAM, which exhibit temperature-responsive behavior, and starch-based materials demonstrate the potential for naturally biocompatible and stimuli-responsive WGMRs, paving the way for adaptive sensing platforms in biological contexts.^85^ The ability to integrate these polymers with inorganic cores, including silica, through composite designs, e.g., (polymer shell)-(inorganic core) WGMRs, further enhances sensing capabilities while maintaining essential biocompatibility.^49,64^ Future research is expected to continue optimizing fabrication techniques and exploring novel biocompatible polymer compositions to enhance sensitivity and expand the application domains of WGMRs in healthcare diagnostics and therapeutic monitoring.

In the present review, we exemplified all-polymer WGMRs displaying promising results in biochemical sensing, environmental monitoring, and integrated photonics. Future developments may presumably focus on improving their sensitivity by optimizing fabrication techniques, enhancing surface functionalization strategies, and exploring new polymer materials with advanced optical properties. Future innovations in all-polymer WGMRs may concentrate on enhancing their sensitivity and functionality in at least four ways. (i) Continued optimization of fabrication techniques and exploration of advanced polymer materials will strive to enhance the sensitivity of all-polymer WGMRs. That includes refining surface functionalization methods and integrating the WGMRs with novel nanomaterials for enhanced light–matter interaction, thus improving the LODs of all-polymer WGMRs. These advancements aim to detect analytes at ultralow concentrations, which is critical for applications in environmental pollution monitoring and medical diagnostics. (ii) Efforts will be made to miniaturize single all-polymer WGMRs and integrate them with microfluidics, microelectronics, and wireless communication technologies for devising portable and POC multimodal sensing tools suitable for field deployment. This approach combines optical, mechanical, and chemical sensing modalities to provide comprehensive analytical capabilities in a compact device. (iii) Advances in polymer coatings and microfluidic integration will enable real-time monitoring of dynamic processes. All-polymer WGMRs equipped with responsive polymer coatings can detect changes in environmental conditions or biomolecular interactions in real time, facilitating timely interventions or data-driven decisions. (iv) Finally, further exploration of biocompatible polymers and their integration into all-polymer WGMRs holds promise for biomedical diagnostics and therapeutic monitoring. Research will presumably focus on improving biocompatibility, stability in physiological environments, and compatibility with existing medical devices.

Moreover, herein, we have discussed polymer film-coated optical-fiber WGMRs, which integrate the advantages of both OFs and polymers to fabricate highly sensitive sensors. Therefore, polymers are beneficial in this hybrid approach. Recent advancements in this field include the development of polymer nanocomposites for enhanced sensitivity and the integration of microfluidic channels for on-chip sample handling.^20^ Future directions in polymer film-coated optical-fiber WGMRs are poised to improve the durability of polymer coatings, thus stabilizing and expanding wavelength ranges for multimodal sensing and integrating wireless communication for remote sensor networks under harsh environmental conditions.^96^ At least the following three directions are envisioned. (i) Particularly, the envisioned research will focus on developing coatings resistant to mechanical wear, harsh chemical exposure, and high-temperature fluctuations. Furthermore, (ii) the expectation is that expanding the wavelength range and broadening sensing modalities of polymer film-coated optical-fiber WGMRs will enable multimodal sensing capabilities.^96^ That includes the integration of polymer film-coated optical-fiber WGMRs with different light sources, detection schemes, and environmental sensors to enhance versatility and applicability. Finally, (iii) the successful integration of polymer film-coated optical-fiber WGMRs with wireless communication technologies and Internet-of-Things (IoT) platforms will enable the operation of autonomous sensor networks.^96^ This integration will facilitate real-time data transmission, remote monitoring, and decision-making in diverse applications, from infrastructure monitoring to healthcare.^97^

Finally, (polymer shell)-(inorganic core) composite-functionalized WGMRs combine the benefits of inorganic materials (including silica and silicon) with polymers to acquire desired optical and mechanical properties. Research in this area is progressing toward devising hybrid materials with optimized RIs and enhanced biocompatibility for biomedical applications.^98^ Future directions in devising inorganic-polymer composite-functionalized WGMRs may include exploring new composite materials, improving fabrication precision, and integrating these resonators into scalable sensor networks for real-time monitoring. In detail, (i) exploring novel advanced composite materials with tailored optical properties and enhanced mechanical stability will expand the applicability of WGMRs in diverse sensing environments. Research efforts will most likely focus on optimizing materials synthesis techniques and characterizing their performance under various conditions. New composite materials will feature optimized RIs, improved sensitivity, enhanced biocompatibility, and decreased interference from external factors, thereby enhancing the reliability of sensing measurements.^96,99^ (ii) Advancements in fabrication precision will possibly enable the production of resonators of complex geometries and integrated photonic circuits. These techniques aim to improve device reproducibility, reliability, and scalability for commercial deployment.^96,100^ (iii) Integrating inorganic-polymer composite-functionalized WGMRs with IoT platforms and cloud-based analytics will undoubtedly facilitate real-time data processing. This integration supports innovative city initiatives, environmental monitoring networks, and personalized healthcare applications.^97^ Furthermore, (iv) functionalizing inorganic-polymer composites with biocompounds or bioactive agents will expand their utility in biomedical sensing. These functionalized resonators will be able to determine biomarkers or pathogens with high selectivity, thus advancing their applications in healthcare diagnostics and therapeutic monitoring.^100^ Lastly, (v) manufacturing inorganic-polymer composite-functionalized WGMRs integrated into scalable sensor networks will surely address real-time and high-throughput data analytics. Using these networks will allow continuous surveillance of environmental parameters or infrastructure integrity, thus supporting proactive maintenance and management strategies.^96,100^

## Conclusions and Future Prospectives

Conclusively, polymers have revolutionized WGMR technology by offering unique advantages in terms of flexibility, tailorability, and biocompatibility. All-polymer WGMRs, polymer film-coated optical-fiber WGMRs, and inorganic-polymer composites-functionalized WGMRs pave avenues for high-sensitivity optical sensing applications. Future developments will likely focus on enhancing sensitivity and detectability, exploring new polymer compositions, and integrating these devices into advanced sensor networks for diverse applications ranging from healthcare diagnostics to environmental monitoring and beyond.

Polymer WGMRs combine high performance, versatility, and cost-effectiveness, positioning them as key devices for advancing photonics and bioengineering. Their unique properties—ranging from high QFs in compact resonators to enhanced nonlinear optical capabilities and tunable properties—enable a broad range of applications, from optical communication to biosensing. As fabrication techniques continue to develop, the potential of polymer WGMRs will only expand, driving innovation across multiple disciplines.

Polymer-based WGMR sensors, crossed at the intersection of photonics, materials science, and sensing technology, are advancing toward achieving ultrahigh sensitivity and selectivity in applications extending from biomedical diagnostics and environmental pollution monitoring to industrial process control. By taking advantage of the unique properties of polymers and WGMs, WGMR sensors offer unparalleled sensitivity and versatility in detecting and quantifying many analytes. Continued research and development efforts are expected to enhance their performance further, expand their application domains, and pave the way toward next-generation optical sensing platforms. Future developments will likely focus on optimizing sensor performance through enhanced fabrication techniques, novel polymer compositions, and integration with advanced signal processing algorithms for real-time data analysis.

## Vocabulary Section

Whispering gallery mode (WGM): is the optical mode that circulates within a resonator, typically of a spherical or cylindrical geometry, due to continuous total internal reflections at the boundary.
The quality factor (QF): a key parameter describing the efficiency of light confinement within the resonator, is the ratio of the energy stored to the energy lost per cycle. A high QF indicates that the resonator can trap light for longer durations, allowing for its multiple circulations around the cavity with minimal energy loss.
An all-polymer whispering gallery mode resonator (WGMR): is a resonator entirely fabricated from polymeric materials. Typically, they are manufactured by spin-coating, soft lithography, or direct laser writing.
A (polymer shell)-(inorganic core) WGMR composite: is a composite that is built of an inorganic core (typically silica) coated with an organic polymer shell. Notable examples include silica microresonator (MR) cores coated with poly­(ethylene glycol) (PEG) or polydimethylsiloxane (PDMS) shells.
Biosensing and chemosensing: indicate analyte sensing using natural (biological) and artificial (synthetic), respectively, receptors.
Free spectral range (FSR): is the spacing in wavelength or frequency between two adjacent resonant modes of a whispering gallery mode resonator. It is inversely proportional to the resonator’s optical path length, determining the spectral resolution.
Refractive index unit (RIU): quantifies the change in refractive index detected by an optical sensor. Sensitivity in nm/RIU describes how much the resonance wavelength shifts in response to a unit change in the refractive index of the surrounding medium.
The thermo-optic effect: is the change in the refractive index of a material with temperature. This effect enables the use of optical resonators as temperature sensors.
A molecularly imprinted polymer (MIP): is a synthetic polymer prepared in the presence of a target molecule (template) and a functional monomer; then, the template is removed from the MIP to leave vacant, selective recognition cavities behind. MIPs are used in sensing applications for selective analyte detection and determination.

## Abbreviations

BSA: bovine serum albumin
Cy5: cyanine 5
CRP: C-reactive protein
cTnI-C: cardiac troponin
dc: direct current
DASP^+^: 4-[*p*-(dimethylamino)­styryl]-1-methylpyridinium (cation)
DS: dye@starch
ELISA: enzyme-linked immunosorbent assay
6-FAM: 6-carboxyfluorescein
FD: freeze-drying (treatment)
FEM: finite element method
FITC: fluorescein isothiocyanate
FRET: Fürster resonance energy transfer
FSR: free spectral range
HGMS: hollow glass microsphere
IgG: immunoglobulin G
IoT: Internet-of-Things
LbL: layer-by-layer (transfer)
LC: liquid crystal
LOC: lab-on-a-chip
LOD: limit of detection
μGC: gas chromatographic microsystem
μOFRR: microfabricated optofluidic ring resonator
MIP: molecularly imprinted polymer
MOF: metal–organic framework
MR: microresonator
MS: microsphere
Nd/YAG: neodymium-doped yttrium aluminum garnet (laser)
ND: neutral density (filter)
NP: nanoparticle
OF: optical fiber
OFRR: optofluidic ring resonator
OMBR: optical microbubble resonator
PEG: poly­(ethylene glycol)
POC: point-of-care
PDMS: polydimethylsiloxane
PEG: poly­(ethylene glycol)
PL: photoluminescence
PMMA: poly­(methyl methacrylate)
PNIPAM: poly­(*N*-isopropylacrylamide)
POF: polymer optical fiber
PS: polystyrene
PSA: prostate-specific antigen
QF: quality factor
R6G: rhodamine-6G
RF: radiofrequency (spectrum)
FSR_RF_: radiofrequency free spectrum range
RH: relative humidity
RI: refractive index
RIU: refractive index unit
SiNP: silica nanoparticle
SMF: single-mode (optical) fiber
SU-8: epoxy-based photoresist
SWCNT: single-walled carbon nanotube
TE: transverse electric (mode)
TEC: thermoelectric cooler
TM: transverse magnetic (mode)
TPP: two-photon polymerization
VEGF: vascular endothelial growth factor
VOC: volatile organic compound
WGM: whispering gallery mode
WGMR: whispering gallery mode resonator
